# Natural variation in flavonol accumulation in Arabidopsis is determined by the flavonol glucosyltransferase BGLU6

**DOI:** 10.1093/jxb/erv546

**Published:** 2015-12-29

**Authors:** Hirofumi Ishihara, Takayuki Tohge, Prisca Viehöver, Alisdair R. Fernie, Bernd Weisshaar, Ralf Stracke

**Affiliations:** ^1^Faculty of Biology & CeBiTec, Bielefeld University,33615 Bielefeld, Germany; ^2^Max-Planck-Institute of Molecular Plant Physiology, Am Mühlenberg 1, 14476 Potsdam-Golm, Germany

**Keywords:** *Arabidopsis thaliana*, flavonoid, flavonol glucosyltransferase, glycoside hydrolase-type, natural variation, whole genome association mapping.

## Abstract

Flavonols are colourless secondary metabolites, primarily regarded as UV-protection pigments that are deposited in plants in their glycosylated forms. The glycosylation of flavonols is mainly catalysed by UDP-sugar-dependent glycosyltransferases (UGTs). Although the structures of flavonol glycosides accumulating in *Arabidopsis thaliana* are known, many genes involved in the ﬂavonol glycosylation pathway are yet to be discovered. The flavonol glycoside profiles of seedlings from 81 naturally occurring *A. thaliana* accessions were screened using high performance thin layer chromatography. A qualitative variation in flavonol 3-*O*-gentiobioside 7-*O*-rhamnoside (F3GG7R) content was identified. L*er* × Col-0 recombinant inbred line mapping and whole genome association mapping led to the identification of a glycoside hydrolase family 1-type gene, *At1g60270/BGLU6*, that encodes a homolog of acyl-glucose-dependent glucosyltransferases involved in the glycosylation of anthocyanins, possibly localized in the cytoplasm, and that is co-expressed with genes linked to phenylpropanoid biosynthesis. A causal single nucleotide polymorphism introducing a premature stop codon in non-producer accessions was found to be absent in the producers. Several other naturally occurring loss-of-function alleles were also identified. Two independent *bglu6* T-DNA insertion mutants from the producer accessions showed loss of F3GG7R. Furthermore, *bglu6* mutant lines complemented with the genomic L*er* BGLU6 gene confirmed that *BGLU6* is essential for production of F3GGR7. We have thus identified an accession-specific gene that causes a qualitative difference in flavonol glycoside accumulation in *A. thaliana* strains. This gene encodes a flavonol 3-*O*-glucoside: 6″-*O*-glucosyltransferase that does not belong to the large canonical family of flavonol glycosyltransferases that use UDP-conjugates as the activated sugar donor substrate.

## Highlight

*BGLU6* is a single trait locus that causes natural qualitative variation in the accumulation of flavonol 3-*O*-gentiobioside 7-*O*-rhamnoside in *Arabidopsis thaliana*. *BGLU6* encodes a glycoside hydrolase-type flavonol 3-*O*-glucoside: 6″-*O*-glucosyltransferase.

## Introduction

Flavonols are colourless natural pigments that are among the most abundant ﬂavonoids in plants. Usually they accumulate in the form of mono-, di-, or triglycosides. Glycosylation enhances the solubility of flavonols and thus is likely to be critical for their transport to and storage in their final destination in the vacuole or cell wall. There is evidence that their biological roles differ across plant species, given that they have, for example, an important role in the maintenance of fertility in *Zea mays* and *Petunia hybrida* (Mo *et al.*, 1992; van der Meer *et al.*, 1992) but not in *Arabidopsis thaliana* or *Eustoma grandiﬂorum* (Burbulis *et al.*, 1996; Nielsen *et al.*, 2002). Because flavonol glycosides absorb UV-B light in the region of 280–320nm, they are regarded as effective UV filters with a major function in tissue protection (Ormrod *et al.*, 1995; Olsson *et al.*, 1998; Solovchenko and Schmitz-Eiberger, 2003). Additionally, ﬂavonols act as UV ﬂower pigments that confer both attractive and defensive functions to insects (Gronquist *et al.*, 2001). They also function as co-pigments, sandwiched between anthocyanin molecules, to modify the colour of ﬂowers and fruits (Goto and Kondo, 1991; Andersen and Jordheim, 2006). Auxin retention and transport, as well as subsequent physiological processes dependent on auxin movement, are also modulated by the accumulation of ﬂavonols (Peer *et al.*, 2011 and references therein; Yin *et al.*, 2014). However, in spite of the obvious importance of ﬂavonols for the plant, the relationship between their structure and function remains unclear (Pollastri and Tattini, 2011 and references therein).

**Fig. 1.**
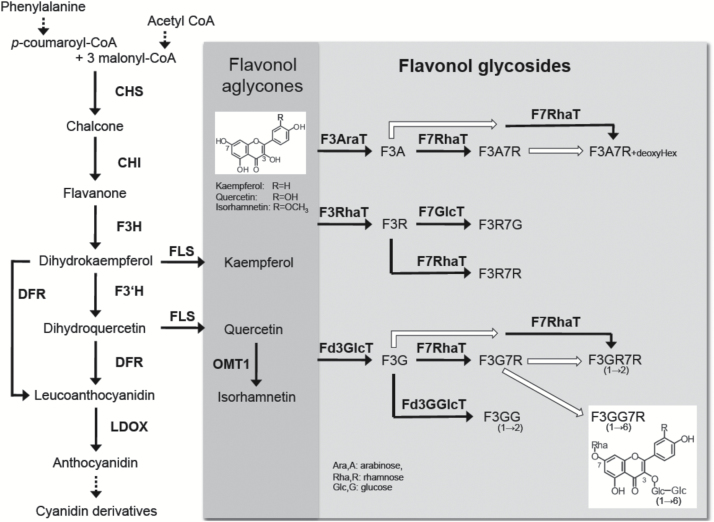
Proposed ﬂavonol biosynthesis and modiﬁcation pathway in *A. thaliana*. The compounds detected in *A. thaliana* are given. White arrows indicate proposed but unidentiﬁed reactions. The metabolite flavonol 3-*O*-gentiobioside 7-*O*-rhamnoside (F3GG7R), which is in the focus of this paper, is shown in the white box. CHI, chalcone isomerase; CHS, chalcone synthase; DFR, dihydroflavonol reductase; F3AraT, ﬂavonol 3-*O*-arabinosyltransferase; F3H, flavanone 3-hydroxylase; F3RhaT, ﬂavonol 3-*O*-rhamnosyltransferase; F3′H, flavonoid 3′-hydroxylase; F7GlcT, ﬂavonol 7-*O*-glucosyltransferase; F7RhaT, ﬂavonol 7-*O*-rhamnosyltransferase; Fd3GGlcT, flavonoid 3-*O*-glucoside glucosyltransferase; Fd3GlcT, ﬂavonoid 3-*O*-glucosyltransferase; FLS, flavonol synthase; LDOX, leucoanthocyanidin reductase (anthocyan synthase); OMT1, *O*-methyltransferase1. Modified according to Saito *et al.* (2013) and Stracke *et al.* (2010), and incorporating data from Yonekura-Sakakibara *et al.* (2014).

Flavonol synthesis is regulated by a variety of developmental and environmental cues (Winkel-Shirley, 2002). The expression of genes in this pathway is light dependent (Pelletier and Shirley, 1996; Cain *et al.*, 1997; Stracke *et al.*, 2010) and regulated by both light quality and quantity through distinct photoreceptors (Wade *et al.*, 2001; Hemm *et al.*, 2004). The expression of flavonol biosynthesis genes is controlled in organ- and tissue-specific patterns by at least three R2R3-MYB transcriptional activators in *A. thaliana* that function without a basic helix-loop-helix partner (Stracke *et al.*, 2007; Stracke *et al.*, 2010). Production of flavonol glycoside 1 (PFG1, MYB12), PFG2 (MYB11), and PFG3 (MYB111) activates, in parallel, the biosynthetic enzyme encoding genes *CHALCONE SYNTHASE* (*CHS*), *CHALCONE ISOMERASE* (*CHI*), *FLAVANONE 3-HYDROXYLASE* (*F3H*), and *FLAVONOL SYNTHASE 1* (*FLS1*) (Mehrtens *et al.*, 2005; Stracke *et al.*, 2007). The enzymes encoded by these four genes, together with the enzyme flavonoid 3′-hydroxylase (F3′H), which converts dihydrokaempferol to dihydroquercetin, are required for the formation of the core ﬂavonol aglycone (Fig. 1). The accumulation of distinct ﬂavonol glycosides is mediated by speciﬁc glycosyltransferases (GTs) (Winkel-Shirley, 2001).

Several *A. thaliana* ﬂavonol GTs have been identiﬁed by transcriptome co-expression- and genome-wide similarity-analyses and were functionally characterized, mainly by studying loss-of-function mutants and substrate analyses of recombinant proteins. This lead to the identification of genes encoding a ﬂavonol 3-*O*-rhamnosyltransferase (F3RhaT, UGT78D1) as well as a ﬂavonol 7-*O*-glucosyltransferase (F7GlcT, UGT73C6) (Jones *et al.*, 2003), a ﬂavonoid 3-*O*-glucosyltransferase (F3GlcT, UGT78D2) (Tohge *et al.*, 2005), a ﬂavonol 7-*O*-rhamnosyltransferase (F7RhaT, UGT89C1), a ﬂavonol 3-*O*-arabinosyltransferase (F3AraT, UGT78D3) (Yonekura-Sakakibara *et al.*, 2007; Yonekura-Sakakibara *et al.*, 2008), a flavonoid 3-*O*-glucoside 2″-*O*-glucosyltransferase (Fd3GGlcT, UGT79B6) (Yonekura-Sakakibara *et al.*, 2014), and a recombinant GT that can utilize ﬂavonol 3-*O*-glucosides as substrate (UGT79B1) (Yonekura-Sakakibara *et al.*, 2012). Glycosylation of flavonols is catalysed by UDP-sugar-dependent glycosyltransferases (UGTs) that are active in the cytosol (Yonekura-Sakakibara and Hanada, 2011). However, even in the highly studied model plant *A. thaliana*, we remain some distance from comprehensively deﬁning the complete ﬂavonol glycoside biosynthesis pathway. The known ﬂavonol structures and the likely presence of unidentiﬁed minor ﬂavonol glycosides suggest that there are as yet unidentiﬁed genes encoding modiﬁcation enzymes.

In *A. thaliana*, a single qualitative trait locus (QTL) on chromosome 1 has been described that corresponds to different glycosylated flavonols (Keurentjes *et al.*, 2006). This QTL was mapped to 88.6 cM using a recombinant inbred line (RIL) population derived from a cross between the accessions L*er* and Cvi. RILs carrying the L*er* allele at this locus accumulate flavonols containing dihexosyl glycosides, whereas lines carrying the Cvi allele at this position do not. The authors speculate that a GT catalysing the production of flavonol dihexosides seems to be active or present in L*er* but not in Cvi, thus affecting the flavonol composition. Alternatively, however, the QTL could encode a regulator of such a structural gene. The same qualitative trait was described by Stracke *et al.* (2009) between the accessions Col-0 and Nö-0. The flavonol derivatives accumulating in Nö-0 seedlings, but not in Col-0 seedlings, have been structurally identified as kaempferol 3-*O*-gentiobioside 7-*O*-rhamnoside (K3GG7R) and quercetin 3-*O*-gentiobioside 7-*O*-rhamnoside (Q3GG7R) (Fig. 1), also named kaempferol/quercetin 3-*O*-beta-[beta-D-glucosyl(1→6)D-glucoside]-7-*O*-alpha-L- rhamnoside (Stracke *et al.*, 2009), which were first described as accumulating in Arabidopsis ‘Wassilewskija-2’ leaves (Veit and Pauli, 1999).

Here, we describe the mapping of this QTL using L*er* × Col-0 RILs and whole genome association mapping. We identify the causal polymorphisms and the protein encoded by the affected locus *BETA GLUCOSIDASE 6* (*BGLU6*). We confirm that *BGLU6* is essential for the production of F3GG7R using knockout mutants and their complementation with a genomic fragment. Thus, in this study, we identify an accession-specific gene that leads to a qualitative difference in flavonol glycoside accumulation in *A. thaliana*, and encodes a newly identified type of flavonol glucosyltransferase that does not belong to the large canonical family of UGTs.

## Material and methods

### *A. thaliana* accessions, mapping population, and insertion mutants

Seeds of all used natural accessions of *A. thaliana* were obtained from the European Arabidopsis Stock Centre (NASC): Nordborg collection (N22660; Supplementary Table S1), Ws-4 (N5390). A RIL population of extensively genotyped lines [100 lines (N1899), 200 lines (N4858)] derived from a cross between the accessions Col-0 and L*er* (Lister and Dean, 1993) was used for QTL mapping (http://arabidopsis.info/CollectionInfo?id=45). T-DNA insertion lines for *At1g60270/BGLU6* were obtained from INRA Versailles (*bglu6-1*, line FLAG_338H01 in Ws-4) and NASC (*bglu6-2*, JIC SM line GT_5_84554 with stock ID N162904 in L*er*). Homozygous insertion lines were identified by PCR-based genotyping. In FLAG_338H01, the wild-type allele was detected with primers RS1252 (5′-TAAGACCTCTCGTCATATCCACTCG-3′) and RS1224 (5′-TTGTGTGACGAAGAAACAAAGTATATGG-3′), and the insertion allele with primers RS1233 (5′-GAAGATGTGAA GTTGATGGTGGACA-3′) and RS1237 (5′-CGTGTGCCAGGT GCCCACGGAATAGT-3′). In GT_5_845554, the wild-type allele was detected with primers RS1233 and RS1228 (5′-CTAGGAGTAA GGAGAGAGGTTGCTCTGC-3′), and the insertion allele with primers RS1228 and B006 (5′-ACGGTCGGGAAACTAGCTCTAC-3′). The resulting PCR products were sequenced to determine the exact insertion positions.

### Plant growth conditions

Surface-sterilized *A. thaliana* seeds were sown on 0.5 Murashige and Skoog medium, on 1.5% sucrose, 0.8% agar plates. They were kept for one day at 4°C in the dark, and then transferred to a phytochamber with 16h of light illumination per day at 21°C and 18.5°C at night. Seedlings were harvested on the 6th day after transfer into the light (5-day-old seedlings).

### Linkage mapping of the F3GG7R trait

The genotype data of 542 molecular markers distributed across all five chromosomes in the Lister and Dean RIL population (Lister and Dean, 1993) was obtained from the European Arabidopsis Stock Center (NASC, http://arabidopsis.info/CollectionInfo?id=45). CAPS makers were generated from the information obtained from Max-Planck Arabidopsis SNP Consortium database (Schmid *et al.*, 2003; Torjek *et al.*, 2003). Additional markers were generated from the L*er* genomic sequence (GenBank AM748036). All additional markers are listed in Supplementary Table S2. New markers and the F3GG7R trait were integrated into the *A. thaliana* genetic map to identify markers significantly linked (base 10 logarithm of odds of 3 and higher) to the F3GG7R trait using Mapmaker/EXP version 3.0b as described in Lander *et al.* (1987).

### Genome-wide association analysis of F3GG7R

The association mapping was performed using the GWAPP web interface, a web application for genome-wide association mapping in Arabidopsis (http://gwas.gmi.oeaw.ac.at) (Seren *et al.*, 2012). The accelerated mixed-model option with default setting was used to identity associations between the F3GG7R phenotype measured from 81 accessions and about 206 000 single nucleotide polymorphisms (SNPs) available from the web interface.

### Flavonol glycoside extraction and detection

Methanolic extracts were produced from 50mg of plant material in 2mL reaction tubes by addition of 0.4mL 80% methanol and about 10–15 zirconia beads of 1mm diameter (Roth). Samples were homogenized in a Precellys 24 homogenizer (Bertin Technologies) at a speed of 6500rpm, two times for 45s. Homogenized samples were incubated for 10min at 70°C and centrifuged for 10min at 15 000g in a standard bench-top centrifuge. Supernatants were vacuum-dried in a SpeedVac at 60°C. The dried pellets were dissolved in 1 µL of 80% methanol mg^−1^ starting material. HPTLC with subsequent diphenylboric acid-2-aminoethyl ester (DPBA) staining of flavonoids was performed according to Wagner and Bladt (1995). Two microliters of methanolic extracts were spotted on 10cm × 10cm silica-60 HPTLC plates (Merck) used as the stationary phase. Adsorption chromatography was carried out using a system of ethyl acetate, formic acid, acetic acid, and water (100:26:12:12, v/v/v/v) as the mobile phase in a closed glass tank. Separated phenylpropanoid compounds were stained by spraying with a 1% DPBA (w/v) (Neu, 1957) solution in methanol, followed by spraying with 5% methanolic polyethylene glycol 4000 (w/v) solution. The stained chromatograms were examined under UV light (312nm) and photographed.

LC-MS was performed as described by Tohge and Fernie. (2010). All data were processed using Xcalibur 2.1 software (Thermo Fisher Scientific). Peak identification and annotation were performed by a combination approach using standard chemical confirmation (Nakabayashi *et al.*, 2009), MS/MS profiling, retention time profiling, and mutant analysis (Tohge *et al.*, 2007; Yonekura-Sakakibara *et al.*, 2008).

### Transcriptome co-expression analysis

The *ATTED-II* database (Obayashi *et al.*, 2007) (version 7.1 from 17 August 2013; http://atted.jp/) was used to identify *BGLU6* co-expressed genes. The strength of gene co-expression is given as Mutual Rank (MR), which is calculated based on the rank of Pearson’s correlation coefficient. Lower MR values indicate stronger gene co-expression.

### RNA isolation and cDNA synthesis

RNA was isolated from 50mg plant material using the NucleoSpin® RNA Plant kit (Macherey-Nagel) according to the manufacturer’s instructions. cDNA was generated from 1 µg total RNA as described previously (Stracke *et al.*, 2007).

### cDNA cloning

The nucleotide sequence of the full-length coding sequence (CDS) of *BGLU6_L*er was obtained by sequencing a *BGLU6_L*er entry construct. PCR was performed with Phusion^®^ polymerase (New England BioLabs) using primers containing attB recombination sites, with cDNAs from 5-day-old *A. thaliana* seedlings as template (primers were RS1253, 5′-attB1-CAATGAAAAAGACTTTTGCTCTGATTACC-3′; and RS1254, 5′-attB2-GGTCGGAGTAAGGAGAGAGGTTGCTCTGC-3′). The resulting amplicon was introduced into the GATEWAY^®^ vector pDONRzeo using BP clonase (Invitrogen).

### Subcellular localization studies

The *BGLU6_L*er CDS containing GATEWAY^®^ Entry plasmid was recombined with the expression vectors pMDC43 (N-terminal green fluorescent protein (GFP) fusion) and pMDC83 (C-terminal GFP fusion) (Curtis and Grossniklaus, 2003), resulting in constructs encoding GFP fusion proteins whose expression is driven by the cauliflower mosaic virus (CaMV) 35S promoter. Bright Yellow 2 (BY2) cells were treated as described in Haasen *et al.* (1999). GFP fluorescence was detected after 20h of incubation in the dark using a Leica DM 5500 B microscope (Leica) equipped with a charge-coupled device camera.

### Complementation experiments with *BGLU6_L*er

A 4050-bp genomic L*er* fragment containing the *BGLU6* gene, including 1217bp upstream of the translation start and 290bp downstream of the stop codon, was amplified with the primers RS1223 (5′-TCGAATAAACCAAATGAAGAAAGACGTG-3′) and RS1224. A corresponding GATEWAY^®^ Entry clone was produced and the genomic *BGLU6* fragment was introduced into the vector pMDC123 (Curtis and Grossniklaus, 2003). The resulting plasmid was transformed into the F3GG7R-negative *A. thaliana* accession Col-0. Flavonol extracts of T2 seedlings of different transgenic lines were analysed by HPTLC and LC-MS.

### Quantitative PCR

Transcript levels of *BGLU6* were determined in triplicate by quantitative PCR using the Platinum^®^ SYBR^®^ Green qPCR SuperMix-UDG (Invitrogen) on a Rotor-Gene 6000 cycler (Corbett Life Science). Gene-specific primers were RS1268 (5′-AGCTGTCTTGGAGTATGTAAAGCA-3′) and RS1269 (5′-ATTTCAGCACACCACCAATGTAAG-3′). As reference gene we used *PEX4* (*PEROXIN4*, *At5g25760*) determined with the use of the primers RS935 (5′-TTGGACGCTTCAGTCTGTGT-3′) and RS936 (5′-TGAACCCTCTCACATCACCA-3′).

### Phylogenetic analysis

BGLU and acyl-glucose-dependent anthocyanin glucosyltransferase (AAGT) protein sequences were aligned by CLUSTALW implemented in MEGA5 (version 5.2.2) (Tamura *et al.*, 2011). A phylogenetic tree was constructed from the aligned protein sequences by MEGA5 using the neighbour-joining method (Saitou and Nei, 1987) with the following parameters: bootstrap method (1000 replicates), Poisson model, uniform rates, and complete deletion.

## Results

### F3GG7R accumulation in *A. thaliana* natural variations

**Fig. 2.**
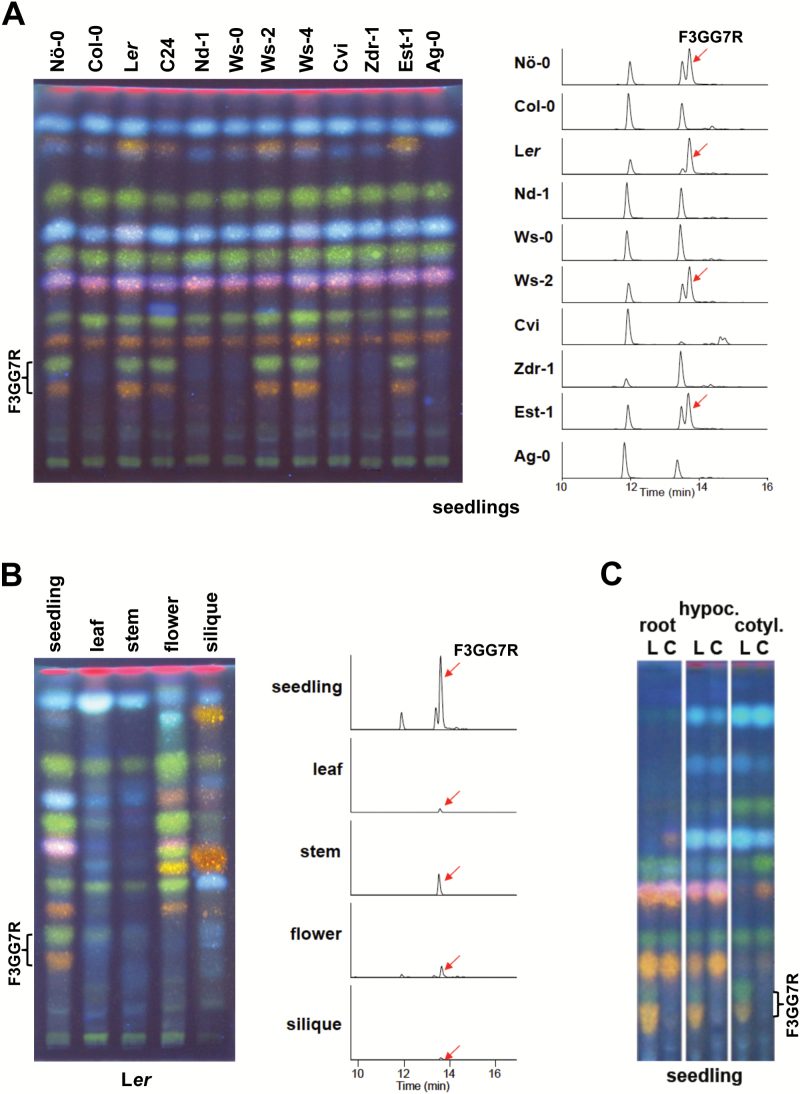
Occurrence of the F3GG7R trait. HPTLC (left) and ion-extracted LC-MS chromatograms, m/z 755 (right) of methanolic extracts. In HPTLC, kaempferol derivatives are identified by green fluorescence, while quercetin derivatives appear yellow/orange. (**A**) Flavonol glycoside accumulation patterns of seedlings from different *A. thaliana* accessions. (**B**) Flavonol glycoside accumulation patterns of L*er* plant organs. (**C**) Flavonol glycoside accumulation patterns of L*er* (L) and Col-0 (C) seedling organs; hypocotyl (hypoc.) and cotyledon (cotyl.).

The observation that the secondary metabolites K3GG7R and Q3GG7R are found in *A. thaliana* seedlings of L*er* and Nö-0 accessions, but not in seedlings of Col-0 and Cvi accessions, led us to question whether they accumulate in other natural accessions. For this purpose we used HPTLC to analyse methanolic extracts of plant material for abundant flavonol glycosides. We used a common staining reagent for flavonoid derivatives, DPBA, which visualizes kaempferol (green) and quercetin derivatives (yellow, orange) under UV illumination. Light-grown seedlings of 81 accessions of the Nordborg ecotype collection (N22660) were analysed by HPTLC; 39 accumulated K3GG7R and Q3GG7R (L*er* phenotype) while the remainder did not (Col-0 phenotype) (Supplementary Table S1). Representative HPTLC results and the corresponding K3GG7R-specific LC-MS chromatograms from a subset of the Nordborg collection are provided in Fig. 2A. The K3GG7R trait always co-occurred with the Q3GG7R trait (and the isorhamnetin-derivative I3GG7R, as demonstrated by LC-MS analyses Supplementary Table S3), indicating that these traits were most likely influenced by a single locus (from now on called the F3GG7R locus/trait). The F3GG7R trait displayed Mendelian inheritance patterns between Col-0 and L*er*, and analysis of F1 seedlings of reciprocal Col-0 × L*er* crosses revealed that the L*er* allele is dominant (data not shown).

### F3GG7R accumulation pattern in *A. thaliana*

Different flavonol derivatives are known to accumulate in distinct organs of *A. thaliana* plants. Given that the patterns of F3GG7R accumulation might provide information about the expression domain(s) of the causal gene, we performed spatial analysis of F3GG7R accumulation. For this purpose, seedlings and adult plants of the L*er* accession were dissected and methanolic extracts were analysed by HPTLC and LC-MS (Fig. 2). Accumulation of F3GG7R compounds was found in all analysed organs of the L*er* seedling (cotyledon, hypocotyl, and root; Fig. 2C), and also in adult plant organs at low levels (Fig. 2B).

### Mapping of the F3GG7R locus by linkage analysis using RILs

Given the dominant nature of the F3GG7R trait, the location of underlying gene in the *A. thaliana* genome could be identified by Mendelian mapping. For this purpose, the F3GG7R phenotypes of 95 L*er* × Col RIL seedlings from the Lister and Dean mapping population (Lister and Dean, 1993) were evaluated using HPTLC analysis, and the seedlings segregated into 32 lines containing the F3GG7R trait (L*er* phenotype) and 63 lines displaying the Col phenotype (Supplementary Table S4). These phenotype scores were used for linkage analysis by comparing the score to the genotype data obtained from 542 polymorphic markers distributed among the five *A. thaliana* chromosomes (http://arabidopsis.info/CollectionInfo?id=45). 

The linkage analysis between the F3GG7R segregation data and the genotype data was carried out using MAPMAKER 3.0b (Lander *et al.*, 1993). The F3GG7R locus was mapped on the lower arm of chromosome 1, tightly linked to the restriction fragment length polymorphism (RFLP) marker mi304 (at 86.52 cM), and with a distance of 0 cM to the anchor markers g4026 (87.02 cM, between 22 276 033 and 22 276 876bp) and nga280 (83.83 cM between 20 873 698 and 20 873 802bp). The interval spans a distance of 1.4 Mbp (Supplementary Fig. S1A). To narrow down the area of the F3GG7R locus, CAPS markers between 22.02 Mbp and 23.82 Mbp on chromosome 1 were generated using information gained from the Max-Planck Arabidopsis SNP Consortium database (Schmid *et al.*, 2003; Torjek *et al.*, 2003). However, the mapping interval could not be shortened due to the lack of recombination events in the interval. By screening 200 additional RILs from the Lister and Dean population, we identified three RILs (N4662, N4674, and N4774) that contained recombination events between the markers. Using additional markers, the interval was reduced down to 106 kbp between the markers H98 (22.2 Mbp) and H238 (22.3 Mbp). A L*er* BiBAC clone (BAC22K22) (Chang *et al.*, 2003) containing a 211-kb L*er* genomic DNA fragment covering the F3GG7R locus was identified and sequenced (GenBank AM748036). Using additional sequence-based markers generated by comparing L*er* and Col-0 genome sequences of the region in question, the F3GG7R locus was located between the markers H238 and H139 (22 270 484bp) with an interval of 87kb (Supplementary Fig. S1B, C). Still, this region contains 29 annotated genes (Supplementary Table S5). In addition, there was no indication that this L*er* region might include an additional gene that is not present in the corresponding Col-0 region.

According to the *Plant Metabolic Network* (http://pmn.plantcyc.org/), K3GG7R should be derived from kaempferol 3-*O*-glucoside 7-*O*-rhamnoside (K3G7R) and UDP-activated glucose, and the reaction should be catalysed by a UGT family-1 protein for which the corresponding gene has not been identified to date. Consequently, we predicted the candidate gene to encode an enzyme involved in UDP-dependent glucosylation of flavonol glycosides. However, examination of the annotation of the 87-kb genome region did not reveal the presence of a UGT-coding gene (Supplementary Table S5).

### Whole genome association mapping of F3GG7R

**Fig. 3.**
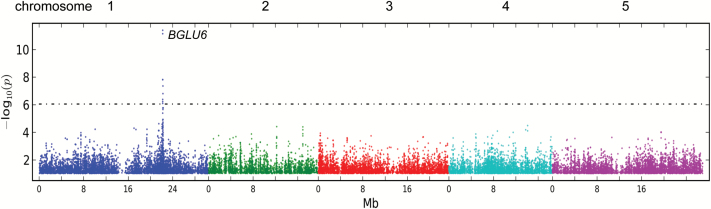
Genome-wide association mapping of F3GG7R. Manhattan plots of genome-wide association mapping results for F3GG7R using 81 accessions from the Nordborg collection. The five *A. thaliana* chromosomes are indicated in different colours. The x-axis indicates the SNP positions in pseudochromosomes. The y-axis indicates the –log10 *p*-value of association with the SNPs. The grey dotted line displays the *p*-value cut off of the 5% false discovery rate. (This figure is available in colour at *JXB* online.)

We performed genome-wide association mapping to identify the genes linked to the F3GG7R phenotype. F3GG7R was measured from 81 accessions from the Nordborg collection and used to identify SNPs associated with the accumulation of F3GG7R. We identified eight SNPs that had significant *p*-values above the cut off of the 5% false discovery rate. All eight SNPs were located between 22 196 382 and 22 267 729bp on pseudochromosome 1. The two highest *p*-values over -log_10_(*p*) = 10 were found on SNPs located at positions 22 222 268bp and 22 222 447bp (Fig. 3) within the gene *BGLU6*, annotated to encode a glycoside hydrolase family 1 (GH1) protein (Miyahara *et al.*, 2011). An earlier study categorized *BGLU6* as a pseudogene because of the lack of key catalytic motifs due to a premature stop codon, which is caused by an SNP located at position 22 222 268bp (Xu *et al.*, 2004). The SNP at position 22 222 447 is located within the ninth intron of *BGLU6*.

**Fig. 4.**
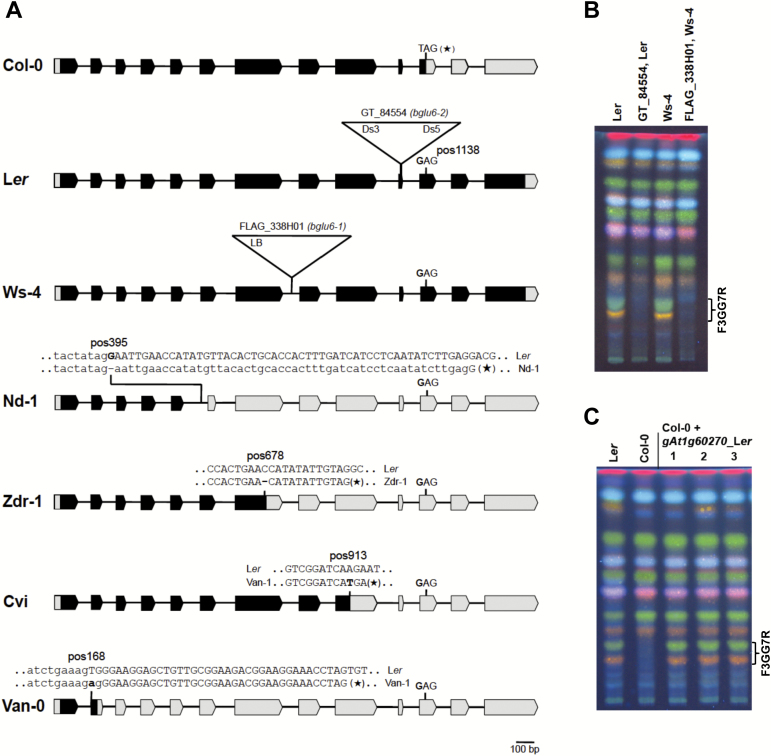
*BGLU6* alleles and their impact on F3GG7R accumulation. (**A**) *BGLU6* alleles. The structure of *At1g60270/BGLU6* wild-type and mutant alleles in different *A. thaliana* accessions and insertion mutants. The exon/intron structure is depicted. Boxed arrows and capital letters indicate exons, while thin black lines and small letters indicate introns. Translated regions are marked in black and untranslated regions in grey. Positions of T-DNA or transposon insertions are given as triangles. SNPs and deletions that are responsible for the loss of function are indicated and their relative position to the start codon (pos) is given. (**B, C**) Flavonol glycoside patterns, HPTLC of methanolic extracts of seedlings. (B) *bglu6* T-DNA insertion mutants. (C) Col-0 complementation lines, carrying a 4.05-kb genomic *BGLU6*_L*er* fragment.

Sequence comparison of the *BGLU6* alleles of Col-0 and L*er* showed that the SNP at 22 222 268bp of pseudochromosome 1 (corresponding to position 1138bp of the *BGLU6* CDS) modifies the stop codon (TAG, present in the Col-0 reference sequence) in the 11th exon into a glutamic acid codon (GAG) (Fig. 4A). Therefore, on the basis of the association data, *BGLU6* is the best candidate gene underlying the F3GG7R trait.

### Naturally occurring *bglu6* alleles

In addition to the above described *bglu6* alleles, which are non-functional because of the SNP at position 22 222 268 (*BGLU6* CDS position 1138), further naturally occurring loss-of-function alleles were identified using the *1001 Genomes* data and confirmed by Sanger sequencing of amplified genomic and cDNA fragments (Fig. 4A, Supplementary Table S1). A deletion of one nucleotide in the seventh exon (*BGLU6* CDS position 678) results in a premature stop codon in the accessions Bor-4, Se-0, Uod-1, and Zdr-1. In Cvi, an SNP in the ninth exon (position 913) alters a glutamic acid codon (GAG) into a premature stop codon (TAG). In two other accessions, splice sites are affected, leading to mis-spliced transcripts. In Nd-1 this is due to a nucleotide deletion at the 5′-splice site of the sixth exon (position 395), and in Van-0 the splice defect is a result of an SNP modifying the 5′-splice site of the second exon (position 168).

The nucleotide variations of *BGLU6* CDSs were compared in the *1001 Genomes* population, currently comprising 504 accessions. SNP 1138 was the most frequent SNP. Of these accessions, 71% display the L*er* allele while only 29% display the Col-0 allele, which causes a premature stop and results in the production of a truncated protein. SNP 1138 has only weak linkage disequilibrium, with three SNPs including silent mutations at positions 819 and 834, and a valine-to-isoleucine exchange of amino acids at position 1177 (Supplementary Table S6). Several SNPs or deletions are unique within the 504 analysed accessions (0.2%); one of them being the already mentioned loss-of-function deletion in Nd-1.

### *BGLU6* is co-expressed with phenylpropanoid biosynthesis genes

Because the Col-0 and L*er BGLU6* promoter sequences are not significantly different (Supplementary Fig. S2), a similar *BGLU6* expression level is assumed in both accessions. We used the *ATTED-II* database (Obayashi *et al.*, 2007) to identify genes transcriptionally co-expressed with *BGLU6*, and to estimate potential functional relationships. Within the 100 best correlating co-expressed *A. thaliana* genes, a striking number of genes related to phenylpropanoid biosynthesis was detected (Supplementary Table S7). These included *TRANSPARENT TESTA6* (*TT6/F3H, At3g51240*), *UGT78D2* (*At5g17050*), *UGT89C1* (*At1g06000*), *4CL3* (*At1g65060*), *UGT75C1* (*At4g14090*), *CHI-like* (*At5g05270*), *UF3GT* (*At5g54060*), *TT19/GST26* (*At5g17220*), *TT5/CHI* (*At3g55120*), *FLS1* (*At5g08640*), *TT3/DFR* (*At5g42800*), *BRT1/UGT84A2* (*At3g21560*), *UGT78D1* (*At1g30530*), and *TT4/CHS* (*At5g13930*). Given that phenylpropanoid metabolism is highly regulated at the transcriptional level, this finding strongly supports the involvement of *BGLU6* in the flavonoid metabolic pathway.

### Identification and analysis of insertional mutants of *bglu6*

Searching the established insertion mutant databases we identified *bglu6* insertion alleles in the F3GG7R-accumulating wild-type accessions L*er* and Ws-4. The T-DNA (FLAG_338H01, Ws-4, *bglu6-1*) and transposon (JIC SM Line GT_5_845554, L*er*, *bglu6-2*) insertions were located in the tenth exon and the seventh intron, respectively (Fig. 4A). Homozygous insertion mutants were identified by PCR and subsequently analysed by HPTLC and LC-MS for F3GG7R accumulation. No insertion mutants displayed accumulation of F3GG7R (Fig. 4B, Supplementary Table S3), indicating two different loss-of-function alleles in different genetic backgrounds. These mutants confirm that a functional *BGLU6* is essential for F3GG7R accumulation.

### An *BGLU6* L*er* fragment complements the Col-0 phenotype

A 4050-bp genomic L*er* fragment containing the *BGLU6* gene, including 1217bp upstream of the translation start and 290bp downstream of the stop codon, was stably introduced in the F3GG7R-negative Col-0 accession via *Agrobacterium*-mediated transformation. Analysis of T2 seedlings of different transgenic lines indicated the accumulation of F3GG7R (Fig. 4C, Supplementary Table S3), confirming that a functional *BGLU6* is required for F3GG7R accumulation.

### *BGLU6* is predominantly expressed in seedlings

**Fig. 5.**
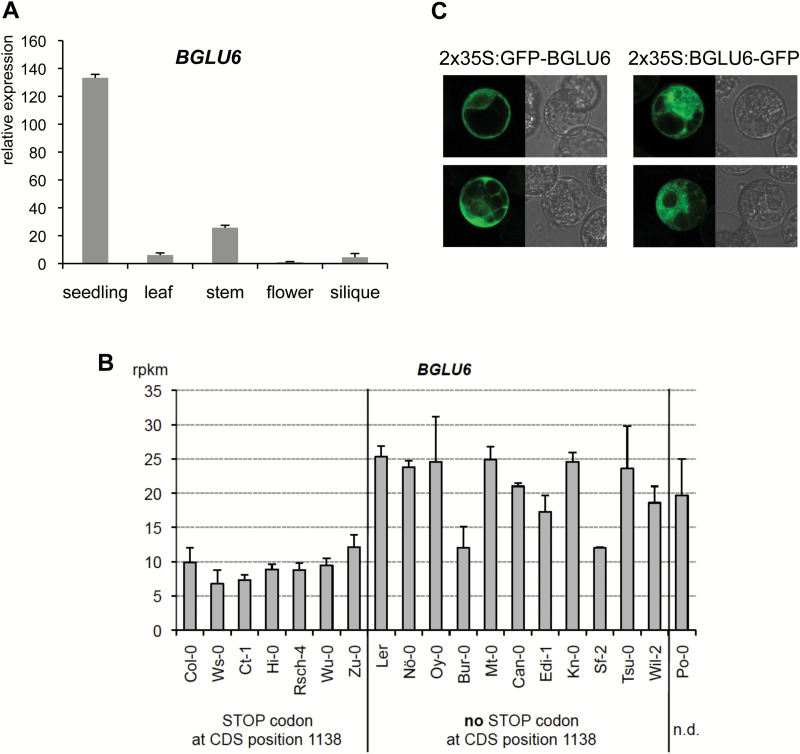
*BGLU6* is mainly expressed in seedlings and the protein seems to be localized in the cytoplasm. (**A**) Quantitative PCR data giving relative *BGLU6* expression in L*er* organs (flower expression level set to 1). (**B**) Expression of *BGLU6* was analysed in seedlings of 19 different *A. thaliana* accessions using RNA-Seq data from the study of Gan *et al.* (2011). Expression levels are given in reads per kilobases per million mapped reads (RPKM). n.d., no data available. Different expression levels between the groups with or without a stop codon at CDS position 1138 are significantly correlated (*p* = 0.000936, Mann–Whitney U-test). (**C**) Subcellular localization of N- and C-terminal GFP fusion proteins with BGLU6 in transiently transfected BY2 protoplasts. Two representative protoplasts are shown for each construct. Left panels: GFP fluorescence. Right panels: bright field image using Nomarski optics. (This figure is available in colour at *JXB* online.)

The expression of *BGLU6* was monitored in L*er* plant organs using quantitative real-time PCR, indicating that *BGLU6* is predominantly expressed in seedlings (Fig. 5A). This expression pattern is in good accordance with the high level of F3GG7R found in the seedling (Fig. 2B).

In addition, we analysed the expression of *BGLU6* in seedlings of 19 different *A. thaliana* accessions using RNA-Seq data from the study of Gan *et al.* (2011). Figure 5B shows that expression levels ranged from 6.8±2.0 to 25.3±1.6 reads per kilobase of exon model per million mapped reads (RPKM). The expression level of *BGLU6* alleles without the premature stop codon (at CDS position 1138) was 20.7±5.0 RPKM (n = 11), while it was 9.0±1.8 RPKM (n = 7) for alleles with a premature stop codon. The different expression levels between the two groups were found to be significantly correlated (*p* = 0.000936, Mann–Whitney U-test). Thus, it seems that the 1138 stop codon may be correlated with a 2.3-times reduction in *BGLU6* expression level in seedlings. The reason for this remains unclear.

### The BGLU6 protein seems to be localized in the cytoplasm

The software *Plant-mPLoc* (Chou and Shen, 2010) predicted that the N-terminal amino acid sequences of BGLU6 would contain a putative transit peptide necessary for localization in the vacuole, and *PlantLoc* (Tang *et al.*, 2013) predicted that BGLU6 would be located in the peroxisome (microbody) or vacuole. To confirm the subcellular localization of BGLU6 protein (encoded by the L*er* allele), N- and C-terminal GFP fusion constructs driven by the CaMV 35S promoter were introduced into tobacco BY2 protoplasts. In contrast to the predictions, a clear GFP signal was observed in the cytoplasm of BY2 cells (Fig. 5C), indicating a cytoplasmic localization of BGLU6-GFP. Although it is possible that the distribution of transiently expressed full-length BGLU6-GFP in BY2 cells might differ from that of the native protein, our results suggest that the cytoplasm is a strong candidate location as the final destination of BGLU6.

## Discussion

Differences in flavonoid composition between naturally occurring *A. thaliana* accessions are mainly quantitative rather than qualitative (Routaboul *et al.*, 2012). However, besides the metabolite F3GG7R described in this work, other accession-specific flavonol glycosides have been reported in leaves of some accessions; for example, kaempferol 3-*O*-rhamnosyl-glucosylglucoside-7-*O*-rhamnoside in C24 (Stobiecki *et al.*, 2006) and kaempferol-hexosylhexoside-deoxyhexoside in Ms-0 (Tohge and Fernie, 2010). *BGLU6* is the first gene to be determined as responsible for the accumulation (or non-accumulation) of an accession-specific flavonol glycoside in *A. thaliana*. The qualitative difference in flavonoids described in this work is not due to the presence of an additional or missing accession-specific gene, but rather to the loss-of-function of a gene present and transcribed in all analysed accessions. The biological function of F3GG7R is currently unknown; however, the accumulation of F3GGR is clearly expendable in many accessions, which could indicate its function in adaptation to different environmental perturbations such as tolerance of high UV radiation. For instance, glycosylation of flavonols has shown to alter absorbance in the UV-B spectral region and suggested to be instrumental in the protection of plants against UV damage (Smith and Markham, 1998). In this respect we tried to correlate F3GG7R production with the level of UV radiation (UV-index) or the altitude at the collection places of the Nordborg *A. thaliana* accessions, but found no obvious correlation (Supplementary Fig. S3). Correlations may be confounded by the underlying structure of the Nordborg collection, given that accessions of this collection are not independent populations, but rather are connected through a genetic and geographical population structure (Weigel, 2012).

From the flavonol glycoside composition in seedlings from the F3GG7R-negative accession Col-0 (Saito *et al.*, 2013), it seems likely that flavonol 3-*O*-glucoside 7-*O*-rhamnoside (F3G7R) is the substrate of BGLU6, which presumably adds a second glucose residue to the present glucose at the 3 position, forming flavonol 3-*O*-glucosyl-glucoside-7-*O*-rhamnoside (Fig. 1). Because these flavonol derivatives are known to have a 1→6 glycosidic linkage between the two glucose residues (Veit and Pauli, 1999; Stracke *et al.*, 2009; Saito *et al.*, 2013), we categorize BGLU6 as a flavonol 3-*O*-glucoside: 6″-*O*-glucosyltransferase. Interestingly, as indicated from the flavonol profile analyses of the *bglu6-1* mutant and *BGLU6* complementation lines (Supplementary Table S3), it seems that the level of the supposed substrate F3G7R is independent of the activity of BGLU6. This may hint to unknown mechanisms that cap the accumulation of flavonol glycosides.

There are some other GTs known to catalyse glycosylation at a sugar moiety attached to flavonol aglycones (FGGTs). For example, UGT707B1 from *Crocus sativa* (saffron) is involved in the formation of kaempferol- and quercetin 3-*O*-glucosyl-(1→2)-glucoside (flavonol 3-*O*-sophorose) (Trapero *et al.*, 2012) and UGT3 from *Catharanthus roseus* (Madagascar periwinkle) catalyses the 1→6-glucosylation of flavonol and flavone glucosides, forming 3-*O*-gentiobioside, gentiotrioside, and gentiotetroside in a sequential manner (Masada *et al.*, 2009). Very recently, *A. thaliana* UGT79B6 was identified as flavonol 3-*O*-glucoside: 2″-*O*-glucosyltransferase, needed for the formation of flavonol 3-*O*-glucosyl-(1→2)-glucoside in pollen (Yonekura-Sakakibara *et al.*, 2014). All three enzymes add a second glucose residue to a flavonol derivative bearing a glucose at the 3 position, forming flavonol 3-*O*-diglucosides with a 1→2 or 1→6 interglycosidic linkage and use UDP-glucose as the activated sugar donor substrate. Similar to CaUGT3, BGLU6 forms a 1→6 interglycosidic linkage, but by contrast shows structural similarity to GH1 group proteins. Because BGLU6 does not belong to the large family of GTs that utilize UDP-conjugates as the activated sugar donor substrate, it represents an atypical type of flavonol glucosyltransferase.

Until recently it had been assumed that glycosylation of flavonoids was catalysed solely by UGTs. In 2010, a novel glucosylation mechanism was reported in flowers of *Dianthus caryophyllus* (carnation) and *Delphinium grandiflorum*, using crude protein extract from plants and purified native and recombinant proteins. The authors found that acyl-glucose-dependent anthocyanin glucosyltransferases (DcAA5GT and DgAA7GT), previously annotated as putative BGLUs, catalysed the formation of anthocyanin 3,5-diglucosides and anthocyanin 3,7-diglucosides using anthocyanin 3-*O*-glucoside and acyl-glucose as substrates (Matsuba *et al.*, 2010). A similar reaction was reported in *A. thaliana* (Miyahara *et al.*, 2013), assigning AAGT activity to the GH1 group protein BGLU10. For AtBGLU10, the energy-rich compound sinapate glucose was identified as the substrate of the glycosylation reaction that leads to the addition of glucose to an acyl moiety (4-coumarate) of an anthocyanin modified with sugars and organic acids.

Based on the sequence similarity of BGLU6 to AtBGLU10 and other AAGTs (Supplementary Fig. S4), we hypothesize that BGLU6 most likely could utilize acyl-glucose derivatives (e.g. glucose esters of sinapic or benzoic acid) rather than UDP sugars as the sugar donors.

In order to test this hypothesis, we tried to examine BGLU6 activity in crude protein extracts prepared from *A. thaliana* L*er* seedlings and to analyse its dependence of flavonoid acceptors and glucose donors. In addition, we tried to characterize BGLU6 activity using an *in vitro* assay with recombinant BGLU6. Unfortunately, both approaches failed. Despite intensive efforts, we could not detect flavonol 3-*O*-glucoside: 6″-*O*-glucosyltransferase activity in L*er* seedling protein extracts and we were unable to achieve high enough yields of active recombinant protein (from expression in *Escherichia coli* and *Pichia pastoris*) to characterize it biochemically. Thus, our functional characterization of the BGLU6 enzyme was limited to that afforded by the expression of this enzyme in a F3GG7R-non-producing *A. thaliana* background (complementation lines, Fig. 4C, Supplementary Table S3), which at least should be sufficient to give a classification as flavonol 3-*O*-glucoside: 6″-*O*-glucosyltransferase for the enzyme’s activity. The question of what the appropriate flavonoid acceptor(s) or glucose donor(s) are remains open.

The amino acid sequences of known GH1-type glucosyltransferases (DcAA5GT, DgAA7GT, AaAA7GT, AtBGLU10) contain the classic highly conserved peptide motifs TFNEP and I/VTENG from GH1 enzymes such as TF/INEA/P and I/VH/L/QENG. The conserved glutamate residues of these key catalytic motifs are not important for distinguishing whether the enzymes are glucosyltransferases or glucosylhydrolases (Matsuba *et al.*, 2010). In BGLU6, the second motif is in accordance with those found in AAGTs; the first motif (TINEG) does not fit, because it has a glycine at the end (Supplementary Fig. S4). Whether this position is related to different substrate specificities (anthocyanins or flavonols) remains to be determined.

**Fig. 6.**
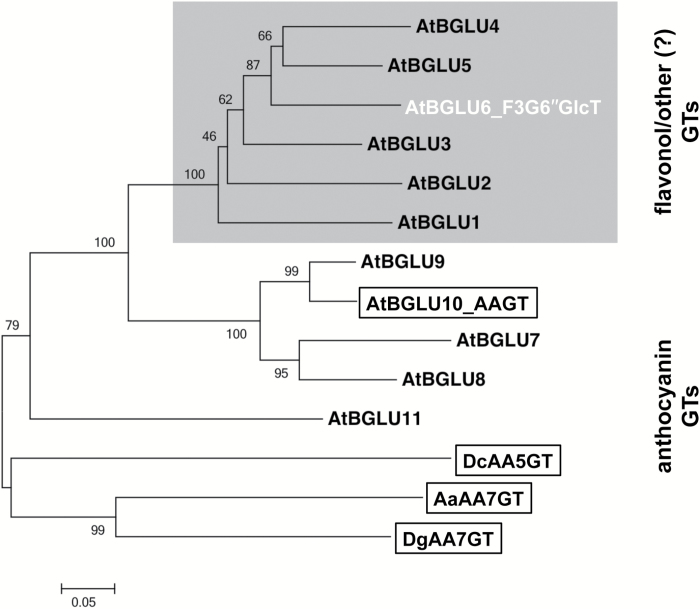
Unrooted molecular phylogenetic neighbour-joining tree of known AAGTs and homologous BGLU proteins from *A. thaliana.* The phylogenetic tree was constructed as described in ‘Methods’. The percentages of replicate trees in which the associated proteins clustered together in the bootstrap test (1000 replicates) are shown next to the branches. Bar = 0.05 amino acid substitutions per site. Known AAGTs are shown boxed and BGLU6 is in white. AtBGLUs showing homology to AAGTs were extracted from Miyahara *et al.* (2011). Abbreviations for species: Aa, *Agapanthus africanus*; At, *Arabidopsis thaliana*; Dc, *Dianthus caryophyllus*; Dg, *Delphinium grandiflorum.*

A phylogenetic analysis of known AAGTs and 11 homologous BGLU proteins from *A. thaliana*, as described by Miyahara *et al.* (2011), indicated a subgroup separated from all proven AAGTs (Fig. 6). Members of this group, comprising AtBGLU1-5 and AtBGLU6, potentially act on substrates other than anthocyanins, as shown for AtBGLU6 in this study. Further work on these AtBGLUs is required to prove this hypothesis.

*BGLU6* co-expression analysis using the *ATTED-II* database returned several genes of flavonoid biosynthesis, including genes encoding (i) the enzymes building the flavonol aglycone, like *CHS*, *CHI*, *F3H*, and *FLS1*; and (ii) the UGTs acting on flavonol substrates like *UGT78D2*/flavonoid 3-*O*-glucosyltransferase, *UGT89C1*/ﬂavonol 7-*O*-rhamnosyltransferase, and *UGT78D1*/ﬂavonol 3-*O*-rhamnosyltransferase. In an alternative approach, *BGLU6* was identified in a study by Miyahara *et al.* (2011) as the third ranked co-expressed gene (by Pearson’s correlation coefficient) using a set of genes that encode enzymes involved in anthocyanin modification as baits, namely *UGT78D2* (At5g17050), *UGT75C1* (At4g14090), and *At5MAT* (At3g29590). These findings clearly support the functional inclusion of BGLU6 within the flavonoid biosynthesis pathway.

Studies by Meissner *et al.* (2008) indicated that the four *A. thaliana UGT84A* genes (*UGT84A1*–*A4*) encode GTs converting hydroxycinnamates to 1-*O*-glucose esters. *UGT84A* expression is induced by UV-B radiation and transgenic *A. thaliana* lines overexpressing single *UGT84A* genes show increased levels of sinapoyl glucose in seeds and seedlings. UGT84A2, which is co-expressed with BGLU6 (Supplementary Table S7), was identified as the main contributor to the production of 1-*O*-sinapoyl glucose (Yonekura-Sakakibara *et al.*, 2012). Under the assumption that the energy-rich, cytosol-located compound sinapoyl glucose (Strack and Sharma, 1985) is a substrate of BGLU6, as it is for other known BGLU-like acyl-glucose-dependent glucosyltransferases (AGTs) (Matsuba *et al.*, 2010; Miyahara *et al.*, 2012; Miyahara *et al.*, 2013), a combined activation of genes forming flavonol glycosides and sinapate glucose would be anticipated to accumulate corresponding substrates. In this respect, it would be interesting to analyse the F3GG7R level in a *ugt84a2* loss-of-function mutant. Unfortunately, to our knowledge, such a mutant allele (natural or artificially inserted) does not exist in a F3GG7R-producing accession. Further analyses will be required to validate the connection between *BGLU6* and *UGT84A* genes.

Because co-expression reflects similar mRNA-level regulation, it is likely that the transcription factors regulating the expression of genes encoding the biosynthesis enzymes also contribute to the establishment of proper conditions (e.g. pH) and compartments (e.g. vacuole, cell wall) for the accumulation of the modified flavonols. The R2R3-MYB transcriptional regulators MYB12, MYB11, and MYB111 (PFG1–3) have been identified as flavonol branch-specific activators, which activate the transcription of biosynthetic enzymes of the general phenylpropanoid pathway (4CL) and the flavonol pathway [CHS, CHI, F3H, FLS, several UGTs (Mehrtens *et al.*, 2005; Stracke *et al.*, 2007; Nakabayashi *et al.*, 2014)]. Specifically, the flavonol-3-*O*-rhamnosyltransferase gene *UGT78D1* and the flavonol 7-*O*-rhamnosyltransferase gene *UGT89C1*, both known to be targets of the PFGs (Stracke *et al.*, 2007), are co-expressed with *BGLU6* (Supplementary Table S7), indicating a possible PFG-dependent concerted expression. In this context we wanted to know if *BGLU6* is a target gene of the PFGs. Using the NewPLACE database (https://sogo.dna.affrc.go.jp/cgi-bin/sogo.cgi?sid=&lang=en&pj=640&action=page&page=newplace), predictive MYB binding sites were found in the 1.2kb *BGLU6* promoter region of 10 natural *A. thaliana* accessions (Supplementary Fig. S2), including *proBGLU6_L*er, which was found to be sufficient for functional complementation (Fig. 4C). To analyse if the three PFGs have the capability to directly bind and activate the *BGLU6* promoter, we used transient co-transfection of *A. thaliana* At7 protoplast cells with 35S-driven PFG1-3 as the effector and a 1.2-kb *BGLU6_L*er promoter–GUS fusion as the reporter. None of the tested R2R3-MYBs were able to activate the *BGLU6* promoter (Supplementary Fig. S5). This result hints that the *BGLU6* promoter itself is not a target of the PFG R2R3-MYBs, like the promoters of the flavonol 3-*O*-arabinosyltransferase *UGT78D3* (Yonekura-Sakakibara *et al.*, 2008) and the flavonol 7-*O*-glucosyltransferase *UGT73C6* (Jones *et al.*, 2003), which were not activated in this assay (Stracke *et al.*, 2010). It at least indicates that *BGLU6* regulation is different from *CHS* regulation. However, a potential activation of these genes *in planta* cannot be formally excluded owing to the possible limitations of the transient co-transfection assay system using hypocotyl-derived At7 protoplasts (Trezzini *et al.*, 1993) or because of missing, unknown co-factors.

We identified BGLU6 as a novel type of flavonol glucosyltransferase not belonging to the large family of GTs that utilize UDP-conjugates as the activated sugar donor substrate. A phylogenetic analysis of all currently known AAGTs and the homologous BGLU proteins from *A. thaliana* revealed two different subgroups (Fig. 6), one containing all AAGTs and a second containing AtBGLU1 to AtBGLU6. Further analyses of AtBGLUs will answer whether this separation is due to different flavonoid substrate specificities.

## Conclusion

In this study we describe a single trait locus causing natural qualitative variation in the accumulation of F3GG7R in *A. thaliana*. This locus was mapped using RILs and whole genome association mapping and found to encode a flavonol 3-*O*-glucoside: 6″-*O*-glucosyltransferase, named BGLU6. Several natural and artificial loss-of-function alleles have been identified, and successful complementation experiments confirmed that functional *BGLU6* is required for F3GG7R accumulation. *BGLU6* was found to be expressed predominantly in the seedling, with the cytoplasm indicated as a candidate for the subcellular localization of the encoded protein. The BGLU6 protein does not belong to the large canonical family of UGT-type flavonol GTs, but represents a GH1-type GT, which have been heretofore only known to act on anthocyanin substrates.

### Accession numbers

*At1g60270/BGLU6*_L*er* gene, cDNA, and protein sequence can be found in the GenBank (NCBI/EMBL/DDBJ) databases under accession number KM047910. The L*er* genomic sequence of the F3GG7R mapped interval can be found in GenBank under accession number AM748036.

## Supplementary material

Supplementary material is available at *JXB* online.

Table S1: Listing of SNPs and deletions (Del) in the *At1g60270* gene of *A. thaliana* natural accessions from the Nordborg collection, correlating with the presence or the absence of F3GG7R accumulation.

Table S2: PCR-based markers. PCR primer sets and restriction enzymes used for genotyping of the RILs are listed.

Table S3: LC-MS derived quantitative flavonol profiles of *A. thaliana* accessions, *BGLU6* T-DNA insertion lines, and *BGLU6* complementation lines.

Table S4: F3GG7R chemotype of Lister and Dean L*er* × Col RILs.

Table S5: List of annotated genes on Col-0 chromosome 1 between the flanking markers H139 and H238.

Table S6: Sequence polymorphisms and their linkage disequilibrium in *BGLU6/At1g60270*.

Table S7: List of 100 genes showing strongest co-expression with *BGLU6*.

Fig. S1: Fine-mapping of the *F3GG7R* locus on chromosome 1.

Fig. S2: *BGLU6* promoter, alignment, and putative MYB binding sites.

Fig. S3: Correlation analyses of F3GG7R production with environmental factors at accession collection places.

Fig. S4: Multiple alignment of GH1-type flavonoid glucosyltransferases.

Fig. S5: Co-transfection analysis of target gene specificities in *A. thaliana* At7 protoplasts.

Supplementary Data
