# Supplementary material for: Natural variation in flavonol accumulation in Arabidopsis is determined by the flavonol glucosyltransferase BGLU6
Source: J Exp Bot. 2015 Dec 29;67(5):1505–17. doi: 10.1093/jxb/erv546 (PMC4762388; doi:10.1093/jxb/erv546)
Supplement: Supplementary Data [file supp_67_5_1505__index.html]

Natural variation in flavonol accumulation in Arabidopsis is determined by the flavonol glucosyltransferase BGLU6 — Supplementary Data 

# Natural variation in flavonol accumulation in Arabidopsis is determined by the flavonol glucosyltransferase BGLU6

## Supplementary Data

Data files

- supplementary\_tables\_S1\_S7\_figures\_S1\_S5.pdf - Supplementary Data
