# Supplementary material for: Natural variation in flavonol accumulation in Arabidopsis is determined by the flavonol glucosyltransferase BGLU6
Source: J Exp Bot. 2015 Dec 29;67(5):1505–17. doi: 10.1093/jxb/erv546 (PMC4762388; doi:10.1093/jxb/erv546)
Supplement: Supplementary Data [file supp_erv546_supplementary_tables_S1_S7_figures_S1_S5.pdf]

Supplementary Data

Natural variation in flavonol accumulation in Arabidopsis is determined by the flavonol glucosyltransferase BGLU6

Hirofumi Ishihara, Takayuki Tohge, Prisca Viehöver, Alisdair R. Fernie, Bernd Weisshaar, Ralf Stracke

Table S1: Listing of SNPs and deletions (Del) in the At1g60270 gene of *A. thaliana* natural accessions from the Nordborg collection, correlating with the presence or the absence of F3GG7R accumulation.

Table S2: PCR-based markers. PCR primer sets and restriction enzymes used for genotyping of the RILs are listed.

Table S3: LC-MS derived quantitative flavonol profiles of *A. thaliana* accessions, *BGLU6* T-DNA insertion lines and *BGLU6* complementation lines.

Table S4: F3GG7R chemotype of Lister and Dean L*er* x Col RILs.

Table S5: List of annotated genes on Col-0 chromosome 1 between the flanking markers H139 and H238.

Table S6: Sequence polymorphisms and their linkage disequilibrium (LD) in *BGLU6/At1g60270*.

Table S7: List of 100 genes showing strongest co-expression with *BGLU6*.

Figure S1: Fine-mapping of the F3GG7R locus on chromosome 1.

Figure S2: *BGLU6* promoter, alignment and putative MYB binding sites.

Figure S3: Correlation analyses of F3GG7R-production with enviromental factors at accession collection places.

Figure S4: Multiple alignment of GH1-type flavonoid glucosyltransferases.

Figure S5: Co-transfection analysis of target gene specificities in *A. thaliana* At7 protoplasts.

Table S1: Listing of SNPs and deletions (Del) in the *At1g60270* gene of *A. thaliana* natural accessions from the Nordborg collection, correlating with the presence (green) or the absence (orange) of F3GG7R accumulation.

The analysed data from the *1001 genomes project* dated from January 2013. Positions of SNPs and Dels are given on *At1g60270* CDS andTAIR10 pseudochromosome 1. Reference accession is Col-0 (marked in red). *contrary to 1001 genomes data, validated by PCR and Sanger sequencing. n.a.: not analyzed.

accession	NASC ID	F3GG7R	1001 genomes data available	SNP 1138 (22,222,268)	Del 678 (22,223,110)	Del 395 (22,223,483)	SNP 913 (22,222,694)	SNP 168 (22,224,061)
Bil-7	N22579	+	+	+	-	-	-	-
Bor-1	N22590	+	+	+	-	-	-	-
C24	N22620	+	+	+ *	-	-	-	-
Est-1	N22629	+	+	+	-	-	-	-
Fab-2	N22576	+	+	+	-	-	-	-
Fab-4	N22577	+	+	+	-	-	-	-
Ga-0	N22634	+	+	+	-	-	-	-
Got-22	N22609	+	+	+	-	-	-	-
Gu-0	N22617	+	+	+	-	-	-	-
Kas-1	N22638	+	+	+	-	-	-	-
Kondara	N22651	+	+	+	-	-	-	-
Ler-1	N22618	+	+	+	-	-	-	-
Lp2-2	N22594	+	+	+	-	-	-	-
Lp2-6	N22595	+	+	+	-	-	-	-
NFA-10	N22599	+	+	+	-	-	-	-
NFA-8	N22598	+	+	+	-	-	-	-
Nok-3	N22643	+	+	+	-	-	-	-
Oy-0	N22658	+	+	+	-	-	-	-
Pro-0	N22649	+	+	+	-	-	-	-
Pu2-23	N22593	+	+	+	-	-	-	-
Pu2-7	N22592	+	+	+	-	-	-	-
Ra-0	N22632	+	+	+	-	-	-	-
Ren-1	N22610	+	+	+	-	-	-	-
RRS-7	N22564	+	+	+	-	-	-	-
Se-0	N22646	+	+	+	-	-	-	-
Sorbo	N22653	+	+	+	-	-	-	-
Ts-1	N22647	+	+	+	-	-	-	-
Ts-5	N22648	+	+	+	-	-	-	-
Tsu-1	N22641	+	+	+	-	-	-	-
Ull2-3	N22587	+	+	+	-	-	-	-
Ull2-5	N22586	+	+	+	-	-	-	-
Uod-7	N22613	+	+	+	-	-	-	-
Wa-1	N22644	+	+	+	-	-	-	-
Ws-2	N22659	+	+	+	-	-	-	-
Sakhdara	N22652	+	-	n.a.	n.a.	n.a.	n.a.	n.a.
CS22491	N22621	+	-	n.a.	n.a.	n.a.	n.a.	n.a.
Var2-1	N22580	+	-	n.a.	n.a.	n.a.	n.a.	n.a.
Zdr-6	N22589	+	-	n.a.	n.a.	n.a.	n.a.	n.a.
**Col-0**	**N22625**	-	+	**reference**	**reference**	**reference**	**reference**	**reference**
Ag-0	N22630	-	+	-	-	-	-	-
An-1	N22626	-	+	-	-	-	-	-
Bay-0	N22633	-	+	-	-	-	-	-
Br-0	N22628	-	+	-	-	-	-	-
CIBC-17	N22603	-	+	-	-	-	-	-
CIBC-5	N22602	-	+	-	-	-	-	-
Ct-1	N22639	-	+	-	-	-	-	-
Ei-2	N22616	-	+	-	-	-	-	-
Fei-0	N22645	-	+	-	-	-	-	-
Gy-0	N22631	-	+	-	-	-	-	-
HR-10	N22597	-	+	-	-	-	-	-
HR-5	N22596	-	+	-	-	-	-	-
Kin-0	N22654	-	+	-	-	-	-	-
Knox-18	N22567	-	+	-	-	-	-	-
LL-0	N22650	-	+	-	-	-	-	-
Mz-0	N22636	-	+	-	-	-	-	-
Pna-10	N22571	-	+	-	-	-	-	-
Pna-17	N22570	-	+	-	-	-	-	-
Ren-11	N22611	-	+	-	-	-	-	-
Rmx-A02	N22568	-	+	-	-	-	-	-
Rmx-A180	N22569	-	+	-	-	-	-	-
RRS-10	N22565	-	+	-	-	-	-	-
Sq-8	N22601	-	+	-	-	-	-	-
Tamm-2	N22604	-	+	-	-	-	-	-
Ws-0	N22623	-	+	-	-	-	-	-
Wt-5	N22637	-	+	-	-	-	-	-
Yo-0	N22624	-	+	-	-	-	-	-
Bor-4	N22591	-	+	+	+	-	-	-
Se-0	N22646	-	+	+	+	-	-	-
Uod-1	N22612	-	+	+	+	-	-	-
Zdr-1	N22588	-	+	+	+	-	-	-
Nd-1	N22619	-	+	+	-	+	-	-
Cvi	N22614	-	+	+	-	-	+	-
Van-0	N22627	-	+	+	-	-	-	+
Bur-0	N22656	-	+	+	-	-	-	-
Eden-2	N22573	-	+	+	-	-	-	-
Lov-1	N22574	-	+	+	-	-	-	-
Lov-5	N22575	-	+	+	-	-	-	-
Mt-0	N22642	-	+	+	-	-	-	-
Knox-10	N22566	-	-	n.a.	n.a.	n.a.	n.a.	n.a.
Kz-1	N22606	-	-	n.a.	n.a.	n.a.	n.a.	n.a.
Mrk-0	N22635	-	-	n.a.	n.a.	n.a.	n.a.	n.a.

Table S2: PCR-based markers.
PCR primer sets and restriction enzymes used for genotyping of the RILs are listed. PCR primers were designed by either comparing Col and L*er* genomic sequence using TAIR L*er* BLAST search or were obtained from MASC (Schmid *et al.*, 2003; Torjek *et al.*, 2003).

For CAPS marker analysis, 5 µL of amplicons, generated by standard PCR with the given primer pairs, were digested with 0.5 units of the appropriate restriction enzyme and 1x restriction enzyme buffer in a total volume of 10 µL in a 96-well PCR plate. The plate was incubated at suitable temperatures for at least 3 h. The lengths of fragments were checked with electrophoresis on a 1% (w/v) agarose gel.

For sequencing markers, amplicons were sequenced with one of the PCR primers. SNPs indicating a L*er* origin are marked in grey.

CAPS marker

CAPS marker	MASC ID	primer (5'->3')	position on chr. 1 [bp]	restriction enzyme
**H206**		H206, ATCAACTCGTCTCTTGTTGCTGCC<br>H207, GACACATCCCCACATAATCAGGAG	21,419,862	*Hin*P1I
**H238**		H238, ATCCCCAGAGGCCAAGTCTAATAG<br>H239, AAAGTGACATGTCCTTATTTTAACTCC	22,184,065	*Eco*RI
**H127**		H127, GGCTAGAACCGAAGTCTCAGG<br>H128, ATCACCATCAAACTTGACCCCC	22,263,044	*Hin*dIII
**H137**		H137, CAATTGCATATTCATTAGGTACCC<br>H138, TTGAAACTTCTCTGGAGTGAAAGG	22,267,814	*Eco*RV
**H98**	MASC02574	H98, GGGTCTGAAGCTATATCGGG<br>H99, GTTATCTGCAGAAGTTTCATCC	22,290,039	*Bmg*I
**H96**	MASC02080	H96, ACAGGTACAGTATTTCATAACATTC<br>H97, ACACACTCTTTGTTCTCGAGATG	22,704,745	*Hyp*188I
**H121**		H121, ATCAATATTCGTGGACGACTAGG<br>H122, AAGCTGGAGACCGTGAACGAC	23,039,704	*Hin*P1I
**H102**	MASC07418	H102, TACAGGGTGCGTAGTTAAACATC<br>H103, CAAAGAAGATAATCATCACTCTCC	23,104,168	AciI

Sequencing marker

marker		primer (5'->3')	position on chr. 1 [bp]
**H309**		H309, CGACACCAGTGGCCATGAAACTCC<br>H310, TATCTGCTGCTGCAAGAACCTACC	22,209,871
	Col-0<br>L*er*	ATTGCTCAACTTCTTTCTCTCATAGGGAGTGGTCTTGGGAAAAATCTCCATCACAAAGT<br>ATTGCTCAACTTCTTTCTCTCATAGGGAGTTGTCTTGGGAAAAATCTCCATCACAAAGT	
**H139**		H139, GTCAGCACCTTAGAGCATCCTC<br>H140, ATGCCGTCTACCTAACTAAATCAC	22,270,466
	Col-0<br>L*er*	AACCAATTATCTCTTAATTAAGAGACAATTTACTTAGGTTTCATCAAATTTAAGAGACT<br>AACCAATTATCTCTTAACTAAGAGACAATTTATCTAGGTTTCATCAAATTTAAGAGACT	

Table S3: LC-MS derived quantitative flavonol profiles of *A. thaliana* accessions, *BGLU6* T-DNA insertion lines and *BGLU6* complementation lines.

***A. thaliana* natural accessions**			Rt	11,88	12,77	12,47	13,59	14,05	13,41	14,71	13,47	14,74	14,28	13,57	14,92	15,31	15,81	16,73	17,06	16,07	17,79	23,75	20,27	23,17
			m/z	755,4	739,5	771,4	755,5	785,3	755,4	739,5	625,4	609,5	579,7	609,6	593,6	623,5	593,6	577,4	607,6	463,3	447,2	461,2	447,3	431,2
			syn.	f8	f3				f8	f3	f26	f21	f25	f6	f2	f14	f5	f1	f30	f16	f18	f28	f24	f17
**sample_ID**	**accession**		FG	**Q3GR7R**	**K3GR7R**	**Q3GG7R**	**K3GG7R**	**I3GG7R**	**Q3GR7R**	**K3GR7R**	**Q3G7G**	**K3G7G**	**Q3A7R**	**Q3G7R**	**K3G7R**	**I3G7R**	**Q3R7R**	**K3R7R**	**I3R7R**	**Q3G**	**K3G**	**I3G**	**Q3R**	**K3R**
141128_Ralf_01	Nö-0	seedlings		2969105	2992443	3388910	5404377	35584	3056207	1825252	0	0	0	11566989	9919328	4487562	3036434	6585967	284694	1831514	490070	73236	677289	758429
141128_Ralf_02	Col-0	seedlings		2296685	6492954	0	0	0	1760500	1904489	0	0	0	13026992	14082677	4729577	5239561	7632012	310500	410030	312672	48126	525567	1178999
141128_Ralf_03	Ler	seedlings		1950350	4096284	1994524	5627993	24648	684070	647763	0	0	0	11129835	10926742	5849896	4477374	9198917	246536	398483	237997	74765	373667	781811
141128_Ralf_04	Ws-2	seedlings		2947407	5391661	4112008	5992969	36196	2630695	1354465	0	0	0	11759620	10981041	5691282	3435753	6848631	245469	262708	118841	87435	633315	748255
141128_Ralf_05	Zdr-1	seedlings		430387	1843083	0	0	0	1813607	1329743	0	0	0	12430220	13628478	4377338	3849765	11029758	223561	388331	169039	68753	617854	1526704
141128_Ralf_06	Nd-1	seedlings		4075763	5906953	0	0	0	3587319	1568829	0	0	0	14087885	14748131	5124067	6499691	8885866	343896	287438	156404	101900	1290122	2396411
141128_Ralf_07	Ag-0	seedlings		2228292	2595290	0	0	0	1261176	494509	0	0	0	11516005	11091734	3998542	2014979	7009556	151825	114978	56829	65935	667006	1072188
141128_Ralf_08	Est-1	seedlings		3465592	6645074	2995657	6185631	368344	3082545	2562668	0	0	0	11998958	12635162	7131099	4037972	9786742	262779	653806	261039	96787	478803	930409
141128_Ralf_09	Bay-0	seedlings		2828703	4333490	0	0	0	2216096	1522260	0	0	0	14065243	15013224	5850633	4588478	11677585	242473	365565	169037	63133	560669	1459125
141128_Ralf_10	Ws-0	seedlings		2327955	7052770	0	0	0	2869948	3624659	0	0	0	14171132	15931463	5481969	3637838	10614542	161317	240093	425537	77498	789440	2429822
141128_Ralf_11	Cvi	seedlings		1707863	4612484	0	0	0	152970	165952	0	0	0	12443385	15711282	5502572	4188319	12310196	254989	948135	796612	62967	411306	1617524
141128_Ralf_12	Ei-2	seedlings		3527772	5101605	0	0	0	2629815	1601461	0	0	0	13169820	13038199	5199568	3353349	10130624	149918	433795	135168	63081	570295	1668157
141128_Ralf_14	Ws-4	seedlings		2074348	4296949	2628033	4489576	32175	1540878	790028	0	0	0	10036366	8866623	4457466	1849362	6399886	143239	227319	131909	93192	605205	739801

bglu6-1 T-DNA insertion mutant				Rt	11,88	12,77	12,47	13,59	14,05	13,41	14,71	13,47	14,74	14,28	13,57	14,92	15,31	15,81	16,73	17,06	16,07	17,79	23,75	20,27	23,17
				m/z	755,4	739,5	771,4	755,5	785,3	755,4	739,5	625,4	609,5	579,7	609,6	593,6	623,5	593,6	577,4	607,6	463,3	447,2	461,2	447,3	431,2
				syn.	f8	f3				f8	f3	f26	f21	f25	f6	f2	f14	f5	f1	f30	f16	f18	f28	f24	f17
**sample_IDs**	**genotype**	**organ**		FG	**Q3GR7R**	**K3GR7R**	**Q3GG7R**	**K3GG7R**	**I3GG7R**	**Q3GR7R**	**K3GR7R**	**Q3G7G**	**K3G7G**	**Q3A7R**	**Q3G7R**	**K3G7R**	**I3G7R**	**Q3R7R**	**K3R7R**	**I3R7R**	**Q3G**	**K3G**	**I3G**	**Q3R**	**K3R**
141128_Ralf_15-17	bglu6-1	seedlings	AVG		1714756	3974325	0	0	0	1227615	678879	0	0	0	9365214	10296735	3890469	1712915	6505512	120210	193783	109483	349258	1735913	3228211
			SE		103827	347966	0	0	0	115672	76941	0	0	0	281159	214998	206453	87519	345210	4275	16515	23260	18228	77917	204017
141128_Ralf_18-20	bglu6-1	inflorescence	AVG		114804	17822600	0	0	0	0	0	2911386	7309166	79286	620003	14767414	2735572	2205956	29494996	1855726	21165	93085	39923	28108	656777
			SE		13227	407125	0	0	0	0	0	241125	132286	9810	74633	537393	142088	121559	390350	101318	1241	2357	3124	1679	59081
141128_Ralf_21-23	bglu6-1	silique	AVG		23307	3761856	0	0	0	0	0	0	0	0	309797	5926734	166813	669569	12984005	162744	371258	12255619	12693	47833	170991
			SE		2187	269072	0	0	0	0	0	0	0	0	58437	383109	15562	63803	530269	8961	19327	267580	548	14599	27794
141128_Ralf_24-26	bglu6-1	stem	AVG		10441	4160927	0	0	0	0	0	0	0	0	17834	3648052	122728	132896	14103759	73369	0	0	0	0	0
			SE		637	81213	0	0	0	0	0	0	0	0	1003	184716	6368	8121	93575	4435	0	0	0	0	0
141128_Ralf_27-29	bglu6-1	rosette leaf	AVG		3312	756248	0	0	0	0	0	0	0	0	7176	440584	19803	22561	2086707	11172	0	0	0	0	0
			SE		1307	184943	0	0	0	0	0	0	0	0	1649	104829	7896	4043	371083	2821	0	0	0	0	0
141128_Ralf_30-32	Ws-4 wt	seedlings	AVG		1709150	4169707	2379899	4983948	359615	1352760	844724	0	0	0	7970962	7988793	3664512	1729967	7213307	145586	169312	104193	343680	1576973	1969069
			SE		221249	501488	294386	411351	48692	246140	150842	0	0	0	801143	771967	441504	294887	436726	23381	21972	16576	9946	70431	118895
141128_Ralf_33-35	Ws-4 wt	inflorescence	AVG		386737	18239919	0	746951	0	0	0	4724940	9150766	186648	1660984	15834654	5993437	4402441	31590160	4127675	42494	149170	89530	64986	1356880
			SE		21922	889835	0	43306	0	0	0	308150	382193	3288	66183	237654	294761	99586	329707	240320	4299	2602	8925	4036	132740
141128_Ralf_36-38	Ws-4 wt	silique	AVG		29554	4787282	0	156974	0	0	0	0	0	0	210855	6331432	273799	594205	13927991	241470	433332	14323172	9325	25667	150573
			SE		1233	214835	0	3693	0	0	0	0	0	0	34264	178331	5393	32861	325426	5655	19726	326219	620	6268	11824
141128_Ralf_39-41	Ws-4 wt	stem	AVG		14258	5061639	0	1249795	49318	0	0	0	0	0	27733	3650159	136826	116967	15490634	84642	0	0	0	0	0
			SE		470	157404	0	56966	1186	0	0	0	0	0	1575	287254	6975	11405	251840	2715	0	0	0	0	0
141128_Ralf_42-44	Ws-4 wt	rosette leaf	AVG		1487	759598	0	326994	0	0	0	0	0	0	8438	168912	16283	11120	2287370	5055	0	0	0	0	0
			SE		329	183018	0	92964	0	0	0	0	0	0	1514	25651	6189	1277	562882	1897	0	0	0	0	0

***BGLU6* complementation lines**				Rt	11,88	12,77	12,47	13,59	14,05	13,41	14,71	13,47	14,74	14,28	13,57	14,92	15,31	15,81	16,73	17,06	16,07	17,79	23,75	20,27	23,17
				m/z	755,4	739,5	771,4	755,5	785,3	755,4	739,5	625,4	609,5	579,7	609,6	593,6	623,5	593,6	577,4	607,6	463,3	447,2	461,2	447,3	431,2
				syn.	f8	f3				f8	f3	f26	f21	f25	f6	f2	f14	f5	f1	f30	f16	f18	f28	f24	f17
**sample_IDs**	**genotype**			FG	**Q3GR7R**	**K3GR7R**	**Q3GG7R**	**K3GG7R**	**I3GG7R**	**Q3GR7R**	**K3GR7R**	**Q3G7G**	**K3G7G**	**Q3A7R**	**Q3G7R**	**K3G7R**	**I3G7R**	**Q3R7R**	**K3R7R**	**I3R7R**	**Q3G**	**K3G**	**I3G**	**Q3R**	**K3R**
141128_hiro_01,13,25	Col-0	rosette leaf	AVG		614879	6109263	0	0	0	0	0	0	0	0	650235	6971349	125241	1464946	8984197	47210	0	0	0	0	0
			SE		84815	735146	0	0	0	0	0	0	0	0	102398	393350	47278	132956	1010807	23571	0	0	0	0	0
141128_hiro_02,14,26	BGLU6 comp 1	rosette leaf	AVG		472730	5134077	413911	4462978	45280	0	0	0	0	0	318794	1444745	68336	1198203	6728269	29079	0	0	0	0	0
			SE		92744	575770	69958	700812	5581	0	0	0	0	0	72166	201037	18526	207647	1025623	6490	0	0	0	0	0
141128_hiro_03,15,27	BGLU6 comp 2	rosette leaf	AVG		491631	6009488	519484	6144697	79039	0	0	0	0	0	316917	1280371	170984	1564528	8479571	117412	0	0	0	0	0
			SE		48767	280691	53716	297390	6827	0	0	0	0	0	65847	135663	77918	168125	982976	52076	0	0	0	0	0
141128_hiro_04,16,28	BGLU6 comp 3	rosette leaf	AVG		211357	3811256	307197	4484398	59538	0	0	0	0	0	142556	590873	84267	969333	5812030	86536	0	0	0	0	0
			SE		22199	474580	29063	462862	9426	0	0	0	0	0	27230	123353	47832	110401	140677	34440	0	0	0	0	0
141128_hiro_05,17,29	BGLU6 comp 4	rosette leaf	AVG		402962	4717213	460436	4525198	61430	0	0	0	0	0	314402	996468	143773	1270712	6527202	92278	0	0	0	0	0
			SE		42258	367427	52275	356802	4071	0	0	0	0	0	72717	213434	23527	197300	660789	23875	0	0	0	0	0
141128_hiro_06,18,30	BGLU6 comp 5	rosette leaf	AVG		553305	7324449	656491	7570244	105610	0	0	0	0	0	248181	841955	153052	1946700	9928472	135663	0	0	0	0	0
			SE		62863	508590	47039	249622	12061	0	0	0	0	0	43896	133426	58607	179357	1110650	38818	0	0	0	0	0

Relative peak area is given. Abbreviations: Rt, retention time; m/z, detected mass; syn., synonym as given in Saito *et al.* 2013; FG, flavonol glycoside; AVG, average; SE, standard error.

Table S4: F3GG7R chemotype of Lister and Dean L*er* x Col RILs.
F3GG7R chemotypes: Col-0 (C), L*er* (L) and no data (n.d.)

NASC ID	*Lister and Dean ID*	*F3GG7R*
N1900	CL4	C
N1901	CL5	C
N1902	CL7	n.d.
N1903	CL13	C
N1904	CL14	C
N1905	CL17	L
N1906	CL19B	L
N1907	CL25	L
N1908	CL29	C
N1909	CL30	C
N1910	CL32	L
N1911	CL33	C
N1912	CL34	C
N1913	CL25	C
N1914	CL36	C
N1915	CL37	C
N1916	CL46	C
N1917	CL52	L
N1918	CL54	C
N1919	CL59	L
N1920	CL62	n.d.
N1921	CL67	C
N1922	CL68	L
N1923	CL71	L
N1924	CL79	C
N1925	CL84	C
N1926	CL90	C
N1927	CL107	C
N1928	CL113	C
N1929	CL115	L
N1930	CL123	C
N1931	CL125	L
N1932	CL131	L
N1933	CL160	C
N1934	CL161	C
N1935	CL166	C
N1936	CL167	C
N1937	CL173	L
N1938	CL175	L
N1939	CL177	C
N1940	CL179	C
N1941	CL180	C
N1942	CL181	L
N1943	CL182	C
N1944	CL188	C
N1945	CL190	L
N1946	CL191	L
N1947	CL193	C
N1948	CL194	L
N1949	CL199	C
N1950	CL209	n.d.
N1951	CL217	L
N1952	CL214	C
N1953	CL231	C
N1954	CL232	C
N1955	CL235	C
N1956	CL237	L
N1957	CL238	C
N1958	CL240	C
N1959	CL242	C
N1960	CL245	C
N1961	CL253	C
N1962	CL257	L
N1963	CL263	C
N1964	CL264	L
N1965	CL266	C
N1966	CL267	L
N1967	CL279	C
N1968	CL283	C
N1969	CL284	L
N1970	CL288	C
N1971	CL295	C
N1972	CL296	L
N1973	CL297	C
N1974	CL302	C
N1975	CL303	L
N1976	CL311	C
N1977	CL321	C
N1978	CL332	C
N1979	CL342	C
N1980	CL345	C
N1981	CL349	n.d.
N1982	CL350	L
N1983	CL351	C
N1984	CL356	L
N1985	CL358	C
N1986	CL359	L
N1987	CL363	C
N1988	CL367	C
N1989	CL370	C
N1990	CL377	C
N1991	CL378	C
N1992	CL386	C
N1993	CL390	C
N1994	CL394	C
N1995	CL395	L
N1996	CL397	L
N1997	CL398	n.d.
N1998	CL400	L
N1999	CL259	L
N4686	CL53	n.d.

Table S5: List of annotated genes on Col-0 chromosome 1 between the flanking markers H139 and H238.
The descriptions of the annotated sequences were from the Arabidopsis genome annotation (TAIR10).

gene ID	*TAIR10 annotation description*
At1g60140	*TPS10 (TREHALOSE PHOSPHATE SYNTHASE 10)* encodes an enzyme putatively involved in trehalose biosynthesis. The protein has a trehalose synthase (TPS)-like domain that may or may not be active as well as a trehalose phosphatase (TPP)-like domain
At1g60150	transposable element gene; pseudogene, similar to putative AP endonuclease/reverse transcriptase
At1g60160	potassium transporter family protein, similar to potassium transporter HAK2p
At1g60170	embryo defective 1220 (emb1220); contains Pre-mRNA processing ribonucleoprotein, snoRNA-binding domain
At1g60180	pseudogene of F-box family protein
At1g60190	PUB19, a plant U-box armadillo repeat protein. Involved in salt inhibition of germination together with PUB18.
At1g60200	splicing factor PWI domain-containing protein / RNA recognition motif (RRM)-containing protein
At1g60220	ULP1D, encodes a deSUMOylating enzyme. In vitro it has both peptidase activity and isopeptidase activity: it can cleave C-terminal residues from SUMO to activate it for attachment to a target protein and it can also act on the isopeptide bond between SUMO and another protein.
At1g60230	radical SHOOT APICAL MERISTEM (SAM) domaincontaining protein
At1g60240	NAC (No Apical Meristem) domain transcriptional regulator superfamily protein
At1g60250	zinc finger (B-box type) family protein
At1g60260	glycosyl hydrolase family 1 protein, *beta glucosidase 5 (BGLU5)*
At1g60270	glycosyl hydrolase family 1 protein, *beta glucosidase 6 (BGLU6)*
At1g60280	NAC023 (Arabidopsis NAC domain containing protein 23); transcription factor; similar to apical meristem formation protein-related
At1g60290	pseudogene, similar to chalcone-flavonone isomerase
At1g60300	Apical meristem formation protein-related
At1g60310	transposable element gene; non-LTR retrotransposon family (LINE)
At1g60320	toll-interleukin-resistance (TIR) domain-containing protein
At1g60330	pseudogene, similar to chalcone-flavonone isomerase
At1g60340	NAC (No Apical Meristem) domain transcriptional regulator superfamily protein
At1g60350	NAC024 (Arabidopsis NAC domain containing protein 24); transcription factor
At1g60360	RING/U-box superfamily protein
At1g60370	F-box family protein
At1g60380	NAC (No Apical Meristem) domain transcriptional regulator superfamily protein
At1g60390	polygalacturonase 1 (PG1)
At1g60400	F-box/RNI-like superfamily protein
At1g60410	a paternally expressed imprinted gene
At1g60420	*NUCLEOREDOXIN 1 (NRX1)*; reduce transmission through pollen

Table S6: Sequence polymorphisms and their linkage disequilibrium (LD) in *BGLU6/At1g60270*.

Positions of *BGLU6* CDS polymorphisms are given in "bp to start codon". Resulting amino acid changes of are indicated at the left. LD (expressed as R^2 value) is classified in six classes (R^2<0.1; 0.1<R^2<0.2; 0.2< R^2<0.4; 0.4< R^2<0.6; 0.6< R^2<0.8; 0.8< R^2) that are shaded from light to dark grey. Minor alleles with allele frequency of 0.1% are excluded from the calculation. For each polymorphism, the allele frequency is counted. LD was calculated using Graphical Genotypes 2.0 (van Berloo *et al.* 2008). Allele frequencies in 504 *A. thaliana* natural accessions are given at the right.

		LD (R^2)																														allele frequency			
BGLU6 variant	bp to Start codon	4	38	55	72	80	179	243	284	387	389	594	649	819	834	1032	1039	1047	1106	1138	1177	1284	1323	1326	1356	1407	1437	1462	1463	1510	1514	Col0 allele	%	non Col0 allele	%
Glu/Lys	4		0,0	0,3	0,8	0,9	0,8	0,7	0,0	0,6	0,7	0,7	0,1	0,2	0,1	0,1	0,5	0,5	0,0	0,2	0,1	0,0	0,3	0,3	0,0	0,3	0,3	0,4	0,3	0,3	0,2	468	92,9	36	7,1
Silent (Ala/Ala)	38	0,0		0,0	0,0	0,0	0,0	0,0	0,0	0,0	0,0	0,0	0,0	0,1	0,1	0,0	0,0	0,0	0,0	0,2	0,1	0,0	0,0	0,0	0,0	0,0	0,0	0,0	0,0	0,0	0,0	495	98,2	9	1,8
Lys/Glu	55	0,3	0,0		0,0	0,4	0,4	0,0	0,0	0,0	0,0	0,2	0,0	0,1	0,1	0,0	0,0	0,3	0,0	0,1	0,1	0,0	0,1	0,8	0,0	0,5	0,5	0,3	0,0	0,3	0,3	497	98,6	7	1,4
Silent (Phe/Phe)	72	0,8	0,0	0,0		0,9	0,8	0,9	0,0	0,8	0,8	0,8	0,1	0,2	0,1	0,1	0,6	0,5	0,0	0,2	0,0	0,0	0,4	0,0	0,1	0,2	0,2	0,2	0,3	0,2	0,1	468	92,9	36	7,1
Cys/Ser	80	0,9	0,0	0,4	0,9		1,0	0,8	0,0	0,7	0,7	0,8	0,1	0,2	0,2	0,1	0,6	0,6	0,0	0,2	0,1	0,0	0,4	0,3	0,1	0,4	0,4	0,3	0,3	0,3	0,2	458	90,9	45	8,9
Arg/Thr	179	0,8	0,0	0,4	0,8	1,0		0,7	0,0	0,7	0,7	0,8	0,1	0,1	0,1	0,1	0,5	0,5	0,0	0,2	0,1	0,0	0,4	0,3	0,1	0,4	0,4	0,3	0,3	0,3	0,2	455	90,3	49	9,7
Tyr/Tyr	243	0,7	0,0	0,0	0,9	0,8	0,7		0,0	0,9	0,9	0,9	0,1	0,2	0,2	0,1	0,7	0,5	0,0	0,2	0,1	0,0	0,5	0,0	0,1	0,2	0,2	0,2	0,3	0,2	0,1	466	92,5	38	7,5
Ala/Gly	284	0,0	0,0	0,0	0,0	0,0	0,0	0,0		0,0	0,0	0,0	0,0	0,2	0,2	0,0	0,0	0,0	0,0	0,1	0,1	0,0	0,0	0,0	0,0	0,0	0,0	0,0	0,0	0,0	0,0	495	98,2	8	1,6
Silent (Val/Val)	387	0,6	0,0	0,0	0,8	0,7	0,7	0,9	0,0		1,0	0,8	0,1	0,1	0,1	0,1	0,7	0,4	0,0	0,2	0,1	0,0	0,5	0,0	0,1	0,2	0,2	0,2	0,3	0,2	0,1	472	93,7	31	6,2
Asn/Ser	389	0,7	0,0	0,0	0,8	0,7	0,7	0,9	0,0	1,0		0,9	0,1	0,1	0,1	0,1	0,7	0,4	0,0	0,2	0,1	0,0	0,5	0,0	0,1	0,2	0,2	0,3	0,3	0,2	0,1	471	93,5	32	6,3
Silent (Asp/Asp)	594	0,7	0,0	0,2	0,8	0,8	0,8	0,9	0,0	0,8	0,9		0,1	0,2	0,2	0,1	0,7	0,5	0,0	0,2	0,1	0,0	0,5	0,1	0,1	0,2	0,2	0,2	0,2	0,1	0,0	463	91,9	41	8,1
Leu/Phe	649	0,1	0,0	0,0	0,1	0,1	0,1	0,1	0,0	0,1	0,1	0,1		0,1	0,1	0,0	0,1	0,0	0,0	0,1	0,1	0,0	0,1	0,0	0,5	0,0	0,0	0,0	0,0	0,0	0,0	482	95,6	22	4,4
Silent (Ala/Ala)	819	0,2	0,1	0,1	0,2	0,2	0,1	0,2	0,2	0,1	0,1	0,2	0,1		1,0	0,1	0,1	0,1	0,1	0,3	0,5	0,1	0,2	0,1	0,0	0,1	0,1	0,1	0,1	0,1	0,1	389	77,2	115	22,8
Silent (Asn/Asn)	834	0,1	0,1	0,1	0,1	0,2	0,1	0,2	0,2	0,1	0,1	0,2	0,1	1,0		0,1	0,1	0,1	0,1	0,3	0,5	0,1	0,2	0,1	0,0	0,1	0,1	0,1	0,1	0,1	0,1	390	77,4	113	22,4
Silent (Ser/Ser)	1032	0,1	0,0	0,0	0,1	0,1	0,1	0,1	0,0	0,1	0,1	0,1	0,0	0,1	0,1		0,1	0,0	0,0	0,1	0,1	0,0	0,6	0,0	0,1	0,0	0,0	0,0	0,0	0,0	0,0	478	94,8	26	5,2
Pro/Thr	1039	0,5	0,0	0,0	0,6	0,6	0,5	0,7	0,0	0,7	0,7	0,7	0,1	0,1	0,1	0,1		0,0	0,0	0,2	0,2	0,0	0,6	0,0	0,1	0,0	0,0	0,0	0,0	0,0	0,0	477	94,6	27	5,4
Silent (Phe/Phe)	1047	0,5	0,0	0,3	0,5	0,6	0,5	0,5	0,0	0,4	0,4	0,5	0,0	0,1	0,1	0,0	0,0		0,0	0,1	0,0	0,0	0,1	0,3	0,3	0,5	0,5	0,6	0,4	0,5	0,3	489	97,0	15	3,0
Asn/Val	1106	0,0	0,0	0,0	0,0	0,0	0,0	0,0	0,0	0,0	0,0	0,0	0,0	0,1	0,1	0,0	0,0	0,0		0,1	0,1	0,0	0,1	0,0	0,0	0,0	0,0	0,0	0,0	0,0	0,0	493	97,8	11	2,2
STOP/Glu	1138	0,2	0,2	0,1	0,2	0,2	0,2	0,2	0,1	0,2	0,2	0,2	0,1	0,3	0,3	0,1	0,2	0,1	0,1		0,4	0,1	0,2	0,1	0,1	0,1	0,1	0,1	0,1	0,1	0,1	145	28,8	359	71,2
Val/Ile	1177	0,1	0,1	0,1	0,0	0,1	0,1	0,1	0,1	0,1	0,1	0,1	0,1	0,5	0,5	0,1	0,2	0,0	0,1	0,4		0,1	0,2	0,0	0,1	0,0	0,0	0,0	0,1	0,0	0,0	347	68,8	157	31,2
Silent (Ile/Ile)	1284	0,0	0,0	0,0	0,0	0,0	0,0	0,0	0,0	0,0	0,0	0,0	0,0	0,1	0,1	0,0	0,0	0,0	0,0	0,1	0,1		0,0	0,0	0,0	0,0	0,0	0,0	0,0	0,0	0,0	499	99,0	5	1,0
Silent (Ser/Ser)	1323	0,3	0,0	0,1	0,4	0,4	0,4	0,5	0,0	0,5	0,5	0,5	0,1	0,2	0,2	0,6	0,6	0,1	0,1	0,2	0,2	0,0		0,0	0,1	0,1	0,1	0,1	0,0	0,1	0,1	444	88,1	60	11,9
Silent (Leu/Leu)	1326	0,3	0,0	0,8	0,0	0,3	0,3	0,0	0,0	0,0	0,0	0,1	0,0	0,1	0,1	0,0	0,0	0,3	0,0	0,1	0,0	0,0	0,0		0,0	0,5	0,5	0,3	0,0	0,3	0,4	498	98,8	6	1,2
Silent (Tyr/Tyr)	1356	0,0	0,0	0,0	0,1	0,1	0,1	0,1	0,0	0,1	0,1	0,1	0,5	0,0	0,0	0,1	0,1	0,3	0,0	0,1	0,1	0,0	0,1	0,0		0,0	0,0	0,0	0,0	0,0	0,0	479	95,0	25	5,0
Silent (Lys/Lys)	1407	0,3	0,0	0,5	0,2	0,4	0,4	0,2	0,0	0,2	0,2	0,2	0,0	0,1	0,1	0,0	0,0	0,5	0,0	0,1	0,0	0,0	0,1	0,5	0,0		1,0	0,9	0,7	0,9	0,8	488	96,8	16	3,2
Silent (Tyr/Tyr)	1437	0,3	0,0	0,5	0,2	0,4	0,4	0,2	0,0	0,2	0,2	0,2	0,0	0,1	0,1	0,0	0,0	0,5	0,0	0,1	0,0	0,0	0,1	0,5	0,0	1,0		0,9	0,7	0,9	0,8	488	96,8	16	3,2
Ala/Thr	1462	0,4	0,0	0,3	0,2	0,3	0,3	0,2	0,0	0,2	0,3	0,2	0,0	0,1	0,1	0,0	0,0	0,6	0,0	0,1	0,0	0,0	0,1	0,3	0,0	0,9	0,9		0,8	0,9	0,8	491	97,4	13	2,6
Ala/Asp	1463	0,3	0,0	0,0	0,3	0,3	0,3	0,3	0,0	0,3	0,3	0,2	0,0	0,1	0,1	0,0	0,0	0,4	0,0	0,1	0,1	0,0	0,0	0,0	0,0	0,7	0,7	0,8		0,7	0,5	495	98,2	9	1,8
Phe/Ser	1510	0,3	0,0	0,3	0,2	0,3	0,3	0,2	0,0	0,2	0,2	0,1	0,0	0,1	0,1	0,0	0,0	0,5	0,0	0,1	0,0	0,0	0,1	0,3	0,0	0,9	0,9	0,9	0,7		0,9	491	97,4	13	2,6
Tyr/Ser	1514	0,2	0,0	0,3	0,1	0,2	0,2	0,1	0,0	0,1	0,1	0,0	0,0	0,1	0,1	0,0	0,0	0,3	0,0	0,1	0,0	0,0	0,1	0,4	0,0	0,8	0,8	0,8	0,5	0,9		494	98,0	10	2,0

Table S7: List of 100 genes showing strongest co-expression with *BGLU6*. The ATTED II database (Obayashi *et al.* 2007; version 7.1 from 17. August 2013; http://atted.jp/) was used to identify *BGLU6* co-expressed genes. The strength of gene co expression is given by Mutual Rank (MR) which is calculated based on the rank of Pearson's correlation coefficient. Lower MR values indicate a stronger gene co expression. Reliability is scored from 0 to 3, with 3 indicating the highest reliability (only genes with reliability scores >0 are given). Genes with (putative) relation to phenylpropanoid biosynthesis pathway are marked in yellow.

Rank	Locus	Alias	(putative) Function	Reliability	MR all	MR (tissue)	MR (abiotic)	MR (biotic)	MR (hormone)	MR (light)
0	At1g60270	BGLU6	beta glucosidase 6		0	0	0	0	0	0
1	At4g34650	SQS2	squalene synthase 2	2	1	70	954,1	14,1	759,2	1027
2	At5g44050	MATE efflux	MATE efflux family protein	1	6,6	63,8	295,6	47,5	58	6190
3	At1g54040	TASTY	epithiospecifier protein	2	7,3	12224,2	2634,3	85,5	167,5	13312
4	At2g40150	TBL28	TRICHOME BIREFRINGENCE-LIKE 28	1	12,1	213,9	299,3	874,7	16917,2	88
5	At4g01080	TBL26	TRICHOME BIREFRINGENCE-LIKE 26	2	15,7	18534,6	6,2	53,9	95,5	11374
6	At5g20070	NUDX19	nudix hydrolase homolog 19	3	16,7	7045,4	12,2	759,3	6330,6	430,3
7	At4g04750	Major facilitator	Major facilitator superfamily protein	2	19,2	541,3	1343,4	9481,4	5312,9	224,2
8	At3g51240	TT6	flavanone 3-hydroxylase	3	20,7	12693,2	30,3	890,5	6631,2	456
9	At4g13410	CSLA15	Nucleotide-diphospho-sugar transferases superfamily protein	2	24	670,7	224,6	13,2	381,6	7791
10	At5g17050	UGT78D2	UDP-glucosyl transferase 78D2	3	26,4	1706,6	18,3	3151,1	7877,7	396,5
11	At1g06000	UGT89C1	UDP-Glycosyltransferase superfamily protein	1	27,4	6724,3	3,5	395,6	5223,1	311,3
12	At5g52320	CYP96A4	cytochrome P450, family 96, subfamily A, polypeptide 4	2	28,4	3519,2	101,9	53,4	16,2	3462
13	At1g65060	4CL3	4-coumarate:CoA ligase 3	3	29,3	9538,5	162,6	4980,1	10775,7	554,9
14	At4g34610	BLH6	BEL1-like homeodomain 6	2	29,5	603,9	67,8	4642,4	4014	237,9
15	At3g48350	CEP3	Cysteine proteinases superfamily protein	2	31,8	8,1	4471,8	10028	50	1153
16	At2g28550	TOE1	related to AP2.7	1	38,2	50,2	137,2	225,6	1091,7	428
17	At1g52000	Mannose-binding lectin	Mannose-binding lectin superfamily protein	3	38,4	1690,6	967,7	138,9	317,3	3441
18	At5g11790	NDL2	N-MYC downregulated-like 2	3	40	2493,7	9,4	120,1	1362,7	638,2
19	At2g39250	SNZ	Integrase-type DNA-binding superfamily protein	1	40,8	934,4	419	521,8	1431,5	1073
20	At2g27420	Cysteineases	Cysteine proteinases superfamily protein	3	42,8	3182,2	478,7	6842,9	8520,6	55,2
21	At2g38750	ANNAT4	annexin 4	1	47,9	645,4	215,9	17,6	133,5	2838
22	At5g55120	VTC5	galactose-1-phosphate guanylyltransferase (GDP)s;GDP-D-glucos	2	49,8	5423,1	97,3	467,7	344	423,1
23	At5g60890	MYB34	myb domain protein 34	1	50,1	20,1	655,3	109,5	57,8	2130
24	At4g14090	UGT75C1	UDP-Glycosyltransferase superfamily protein	1	50,2	60,8	514,2	6696,8	3269,9	77,2
25	At2g36590	ProT3	proline transporter 3	1	52,9	14707,7	3,5	95	1202	238,3
26	At5g05270	isomerase	Chalcone-flavanone isomerase family protein	3	53,1	10337,7	96,7	1088,8	7795,2	882,4
27	At4g17470	alpha/beta-Hydrolases	alpha/beta-Hydrolases superfamily protein	2	54,3	426	2367,6	950,1	123,5	2650
28	At2g40460	Major facilitator	Major facilitator superfamily protein	2	55,2	19776,4	164,5	82,7	195,9	809,1
29	At3g23210	bHLH34	basic helix-loop-helix (bHLH) DNA-binding superfamily protein	3	55,8	5440,3	39	7490	10504	1561
30	At4g04840	MSRB6	methionine sulfoxide reductase B6	1	57,8	914,5	3558,9	265,5	249,7	817,5
31	At1g65860	FMO GS-OX1	flavin-monooxygenase glucosinolate S-oxygenase 1	3	58,9	248,2	245,3	804,2	229,8	12653
32	At4g15440	HPL1	hydroperoxide lyase 1	3	61,1	14488,4	62,9	880,8	59	4825
33	At4g36830	GNS1	GNS1/SUR4 membrane protein family	1	62,1	10650,2	667,2	520,1	616,5	1409
34	At1g78440	GA2OX1	Arabidopsis thaliana gibberellin 2-oxidase 1	2	62,1	8851	1392,3	725,1	5368	256,1
35	At5g09870	CESA5	cellulose synthase 5	3	64	2873,8	7,8	115,9	2265	1034
36	At3g17130	inhibitor	Plant invertase/pectin methylesterase inhibitor superfamily protein	1	64,1	21492,4	663,7	1	4503,8	318,4
37	At3g52370	FLA15	FASCICLIN-like arabinogalactan protein 15 precursor	2	65,8	5037,6	44	178,2	4369,9	686,6
38	At5g03760	RAT4	Nucleotide-diphospho-sugar transferases superfamily protein	2	66,9	3339	79	296,5	271,2	568,1
39	At3g27170	CLC-B	chloride channel B	3	67,8	1744,5	304,2	75,3	279,9	523,2
40	At2g20340	transferase	Pyridoxal phosphate (PLP)-dependent transferases superfamily pr	2	68,1	6872,8	148,9	14,3	155,8	1486
41	At1g48100	Pectin lyase-like	Pectin lyase-like superfamily protein	1	69,7	19658,3	203,8	303	1989,6	4
42	At5g42760	transferase	Leucine carboxyl methyltransferase	3	70,5	13350,9	131,9	4131,1	9880	7,3
43	At2g32990	GH9B8	glycosyl hydrolase 9B8	2	75,6	7164,5	28,3	33	1249,3	413,1
44	At5g54060	UGT79B1	UDP-glucose:flavonoid 3-o-glucosyltransferase	1	76,7	204,5	213,9	5368,7	3267,4	44,2
45	At4g12030	BAT5	bile acid transporter 5	3	78,3	197,5	2751,1	7043,8	1695	19063
46	At1g24070	CSLA10	cellulose synthase-like A10	3	78,7	12197,1	948,7	200,5	224,3	8726
47	At5g67150	transferase	HXXXD-type acyl-transferase family protein	2	82,2	1180,8	2041,2	1493,5	267,4	7278
48	At3g29590	5MAT	HXXXD-type acyl-transferase family protein	2	83,4	104,2	796,5	8064,9	1692,7	202,7
49	At3g03190	GSTF11	glutathione S-transferase F11	3	86,7	50,4	2941,4	661,2	634,5	15767
50	At5g17220	TT19	glutathione S-transferase phi 12	2	87,3	3606,2	241,3	5895,3	8143,8	112,5
51	At3g54990	SMZ	Integrase-type DNA-binding superfamily protein	1	93,9	396,9	143,1	7256,9	613,9	587
52	At1g72500			1	96	967	3672,5	427,4	373,4	5,5
53	At3g02830	ZFN1	zinc finger protein 1	2	97,4	7856,2	82,3	5628,6	5975	82,2
54	At1g10370	GSTU17	Glutathione S-transferase family protein	2	99,4	13288	248,8	978,5	6504,1	281,2
55	At2g24540	AFR	Galactose oxidase/kelch repeat superfamily protein	3	100,4	9733	277,1	698,3	6403,6	319,9
56	At1g60590	Pectin lyase-like	Pectin lyase-like superfamily protein	2	100,6	3737,4	1563,6	12575	3357,3	31,8
57	At1g62560	FMO GS-OX3	flavin-monooxygenase glucosinolate S-oxygenase 3	3	102,4	320,9	105,7	9100	841,3	9354
58	At1g56670	hydrolase	GDSL-like Lipase/Acylhydrolase superfamily protein	1	102,5	7018,7	97,9	307,4	13911,9	14770
59	At1g56650	SIAA1	production of anthocyanin pigment 1	2	105	6939,7	917,2	4975,3	3863	93,9
60	At5g48880	PKT2	peroxisomal 3-keto-acyl-CoA thiolase 2	3	105	16028	453,2	114,5	5855,1	467,2
61	At2g31790	transferase	UDP-Glycosyltransferase superfamily protein	1	105,7	246,6	2861,1	9707,7	1845,8	718
62	At3g55120	TT5	Chalcone-flavanone isomerase family protein	3	106,2	10646,8	116	7559,5	16061,6	844,1
63	At3g17120			1	108,1	1601,8	582,2	1,4	75,5	8599
64	At1g12370	UVR2	photolyase 1	3	109	11825,3	107,4	777,5	8856,5	77,7
65	At4g16140	proline-rich	proline-rich family protein	1	109,3	5324,8	354,8	19,9	75,3	2499
66	At3g21890	zinc finger	B-box type zinc finger family protein	3	112,2	20047,8	114	323,2	4446,3	877,5
67	At1g62540	FMO GS-OX2	flavin-monooxygenase glucosinolate S-oxygenase 2	1	113,7	2406,3	697	7577,3	6970,7	3147
68	At5g20220	CCHC-type	zinc knuckle (CCHC-type) family protein	2	114,1	4437,2	194,9	1287,3	7729,2	9,4
69	At5g07690	PMG2	myb domain protein 29	3	114,6	90,5	2338,4	5955,7	302,5	9462
70	At3g15790	MBD11	methyl-CPG-binding domain 11	1	115	16011,9	282,7	33,6	231,6	2341
71	At5g44110	POP1	P-loop containing nucleoside triphosphate hydrolases superfamily	3	120,4	6435,1	68,1	738,2	8124	1330
72	At4g10390	kinase	Protein kinase superfamily protein	2	120,8	1044,1	80,5	254,2	153,6	1205
73	At2g36870	XTH32	xyloglucan endotransglucosylase/hydrolase 32	2	121,7	786,1	100	1971,5	7736,1	171,2
74	At5g08640	FLS1	flavonol synthase 1	3	122,3	13380,9	56,2	3519,2	11252,1	1909
75	At5g42800	TT3	dihydroflavonol 4-reductase	2	127,3	2252,4	56,3	6585,4	5948,9	858,1
76	At2g38760	ANNAT3	annexin 3	1	128,5	5235,7	482	299,5	83,4	3218
77	At2g44940	DNA-binding	Integrase-type DNA-binding superfamily protein	2	130,1	2813,7	303,1	675,1	170,4	58,7
78	At3g17609	HYH	HY5-homolog	3	130,7	1172,2	67,3	10089	19576,3	147,8
79	At5g48850	SDI1	Tetratricopeptide repeat (TPR)-like superfamily protein	3	131,2	12641,2	7583,5	166,5	6120,2	18438
80	At2g34490	CYP710A2	cytochrome P450, family 710, subfamily A, polypeptide 2	1	132,7	1760,7	242,3	6374,7	15333,8	2281
81	At3g21560	UGT84A2	UDP-Glycosyltransferase superfamily protein	2	134,2	7499,8	138,6	17644	14278	376,5
82	At3g61220	SDR1	NAD(P)-binding Rossmann-fold superfamily protein	2	134,4	7295,4	81,2	11473	12301	79,8
83	At2g39330	JAL23	jacalin-related lectin 23	3	134,5	10683,4	366,6	1061,9	1317,9	6965
84	At2g19780	LRR	Leucine-rich repeat (LRR) family protein	2	135,3	3752,2	766,4	28,1	22,4	18945
85	At5g15740	transferase	O-fucosyltransferase family protein	2	136,7	14428,2	33,8	212,7	19701,9	29,4
86	At2g29450	GSTU5	glutathione S-transferase tau 5	2	138,3	10420,4	2775,5	137,7	70,2	988,5
87	At2g25530	ATPase	AFG1-like ATPase family protein	3	139	18789,3	85,5	1865,9	10607,6	204,9
88	At1g30530	UGT78D1	UDP-glucosyl transferase 78D1	3	141,3	7848,2	40	4825,3	3810,4	27,4
89	At1g19670	CORI1	chlorophyllase 1	2	144	3943,9	1065,5	500,8	134,8	60,8
90	At5g61810	APC1	Mitochondrial substrate carrier family protein	2	145,3	8057,8	3375,4	23,5	42,4	634
91	At1g32900	GBSS1	UDP-Glycosyltransferase superfamily protein	3	146,7	10197,4	1244,7	282,8	15899,7	193,5
92	At3g52740			3	147	10795,9	23,8	405,5	12786,9	68,2
93	At5g23820	MD-2-related lipid recogr	MD-2-related lipid recognition domain-containing protein	3	149,7	6	2006,9	1260,4	1003,8	4380
94	At3g57020	phosphotriesterase	Calcium-dependent phosphotriesterase superfamily protein	1	152,3	3456,3	545,9	898,8	2890,8	805,5
95	At5g62220	GT18	glycosyltransferase 18	2	152,4	6784,5	199,3	1386,9	188,9	20,1
96	At1g18870	ICS2	isochorismate synthase 2	1	158	4648,2	4129,6	709,5	2893,2	3157
97	At4g02850	PhzC/PhzF	phenazine biosynthesis PhzC/PhzF family protein	1	159,7	1	1590,7	3679,2	1051,4	274
98	At4g24010	CSLG1	cellulose synthase like G1	2	159,9	10634,5	419,8	285,3	5197,4	6834
99	At5g13930	TT4	Chalcone and stilbene synthase family protein	3	160,1	10397,4	61	1479,8	11465	1246
100	At1g01520	ASG4	Homeodomain-like superfamily protein	3	160,3	16491,3	300,9	5310,7	4578,6	6145

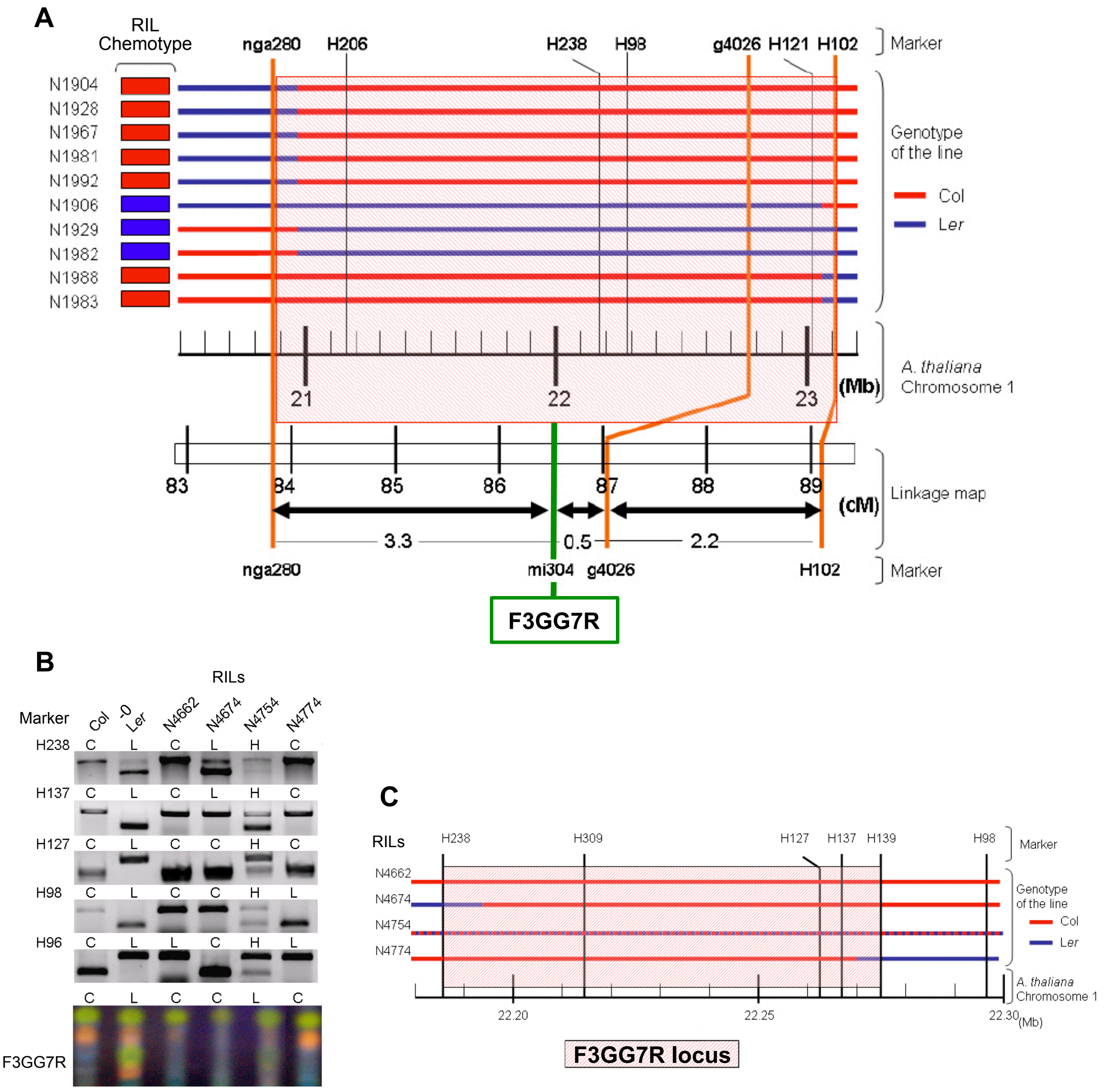

Figure S1: Fine-mapping of the F3GG7R locus on chromosome 1.
(A) The linkage map of the F3GG7R locus area, constructed using the MAPMAKER program, was integrated to the physical map of *A. thaliana* chromosome 1 by anchor markers H102, g4026 and nga280. Chemotypes of the RILs are listed next to line names as Col-0 (red box) and L*er* (blue box). The genotypes of the RILs at the marker positions are indicated with red (Col-0) or blue (L*er*) solid lines. The position of the F3GG7R locus is indicated with a green vertical line at 86.52 cM. The genetic distance (in cM) between the markers and the F3GG7R locus are given below the arrows.
(B) Geno- and phenotypical analysis of selected RILs containing recombination events between the flanking markers H96 and H238. Selected RILs were analysed using CAPS markers and F3GG7R chemotype marker. Genotype key: Col-0 (C), L*er* (L), heterozygous (H).
(C) F3GG7R locus on *A. thaliana* chromosome 1 between the sequence-based map markers H238 (22.18 Mb) and H139 (22.27 Mb).

```
            -1335  AC-rich element
Col-0       ATTTCACCAACATTCCACCGACATATCTCTCTCGCGGGAAAGGGCATTTTAAAAACATTT -1276
Ws-0        ATTTCACCAACATTCCACCGACATATCTCTCTCGCGGGAAAGGGCATTTTAAAAACATTT
Ct-1        ATTTCACCAACATTCCACCGACATATCTCTCTCGCGGGAAAGGGCATTTTAAAAACATTT
Nd-1        ATTTCACCAACATTCCACCGACATATCTCTCTCGCGGGAAAGGGCATTTTAAAAACATTT
Cvi         ATTTCACCAACATTCCACCGACATATCTCTCTCGCGGGAAAGGGCATTTTAAATACATTT
#Nö-0       ATTTCACCAACATTCCACCGACATATCTCTCTCGCGGGAAAGGGCATTTTAAAAACATTT
#Oy-0       ATTTCACCAACATTCCACCGACATATCTCTCTCGCGGGAAAGGGCATTTTAAAAACATTT
#C24        ATTTCACCAACATTCCACCGACATATCTCTCTCGCGGGAAAGGGCATTTTAAAAACATTT
#Bur-0      ATTTCACCAACATTCCACCGACATATCTCTCTCGCGGGAAAGGGCATTTTAAAAACATTT
#Ler        ATTTCACCAACATTCCACCGACATATCTCTCTCGCGGGAAAGGGCATTTTAAAAACATTT

Col-0       AGCAATTTAGGAGCGATGCTCAATATTTATTTATTTTTTTTTTATTTTTTTTGGATGTAA -1216
Ws-0        AGCAATTTAGGAGCGATGCTCAATATTTATTTATTTTTTTTTTATTTTTTT-GGATGTAA
Ct-1        AGCAATTTAGGAGCGATGCTCAATATTTATTTATTTTTTTTTTATTTTTTTTGGATGTAA
Nd-1        AGCAATTTAGGAGCGATGCTCAATATTTATTTATTTTTTTTTTATTTTTTTTGGATGTAA
Cvi         AGCAATTTAGGAGCGATGCTCAATATTTTTTTATTTATTATTTATTTTTTTTGGATGTAA
#Nö-0       AGCAATTTAGGAGCGATGCTCAATATTTATTTATTTATTTTTTATTTTTTTTGGATGTAA
#Oy-0       AGCAATTTAGGAGCGATGCTCAATATTTATTTATTTTTTTTTTTTTTTTTTTGGATGTAA
#C24        AGCAATTTAGGAGCGATGCTCAATATTTATTTATTTTTTTTTTATTTTTTTTGGATGTAA
#Bur-0      AGCAATTTAGGAGCGATGCTCAATATTTATTTATTTTTTTTTTTTTTTTTTTGGATGTAA
#Ler        AGCAATTTAGGAGCGATGCTCAATATTTATTTATTTTTTTTTTATTTTTTTTGGATGTAA

                              MYB core element
Col-0       ATTCAAATATAACACAATAACGGTACTATCATAAATTACTAGCTTACCTATCTGAATTTT -1156
Ws-0        ATTCAAATATAACACAATAACGGTACTATCATAAATTACTAGCTTACCTATCTGAATTTT
Ct-1        ATTCAAATATAACACAATAACGGTACTATCATAAATTACTAGCTTACCTATCTGAATTTT
Nd-1        ATTCAAATATAACACAATAACGGTACTATCATAAATTACTAGCTTACCTATCTGAATTTT
Cvi         ATTCAAATATAACACAATAACGGTACTATCATAAATTACTAGCTTACCTATCTGAATTTT
#Nö-0       ATTCAAATATAACACAATAACGGTACTATCATAAATTACTAGCTTACCTATCTGAATTTT
#Oy-0       ATTCAAATATAACACAATAACGGTACTATCATAAATTACTAGCTTACCTATCTGAATTTT
#C24        ATTCAAATATAACACAATAACGGTACTATCATAAATTACTAGCTTACCTATCTGAATTTT
#Bur-0      ATTCAAATATAACACAATAACGGTACTATCATAAATTACTAGCTTACCTATCTGAATTTT
#Ler        ATTCAAATATAACACAATAACGGTACTATCATAAATTACTAGCTTACCTATCTGAATTTT

Col-0       CATTTTGTTATTTATAAATATAATGGTATTTCATTATATGATATCAAACTTAAAATATCT -1096
Ws-0        CATTTTGTTATTTATAAATATAATGGTATTTCATTATATGATATCAAACTTAAAATATCT
Ct-1        CATTTTGTTATTTATAAATATAATGGTATTTCATTATATGATATCAAACTTAAAATATCT
Nd-1        CATTTTGTTATTTATAAATATAATGATATTTCATTATATGATATCAAACTTAAAATATCT
Cvi         CATTTTGTGATTTATAAATATAATGGTATTTCATTATATGATATCAAACTTAAAATATCT
#Nö-0       CATTTTGTTATTTATAAATATAATGGTATTTCATTATATGATATCAAACTTAAAATATCT
#Oy-0       CATTTTGTTATTTATAAATATAATGGTATTTCATTATATGATATCAAACTTAAAATATCT
#C24        CATTTTGTTATTTATAAATATAATGGTATTTCATTATATGATATCAAACTTAAAATATCT
#Bur-0      CATTTTGTTATTTATAAATATAATGGTATTTCATTATATGATATCAAACTTAAAATATCT
#Ler        CATTTTGTGATTTATAAATATAATGGTATTTCATTATATGATATCAAACTTAAAATATCT

Col-0       AAATAAATATGATTTCTCATAATTTATTTAATCTAAAAATGCGTATGGAAATAACACAAA -1036
Ws-0        AAATAAATATGATTTCTCATAATTTATTTAATCTAAAAATGCGTATGGAAATAACACAAA
Ct-1        AAATAAATATGATTTCTCATAATTTATTTAATCTAAAAATGCGTATGGAAATAACACAAA
Nd-1        AAATAAATATGATTTCTCATAATTTATTTAATCTAAAAATGCGTATGGAAATAACACAAA
Cvi         AAATAAATATGATTTCTCATAATTTATTTAATCTAAAAATGCGTATGGAAATAACACAAA
#Nö-0       AAATAAATATGATTTCTCATAATTTATTTAATCTAAAA-TGCGTATGGAAATAACACAAA
#Oy-0       AAATAAATATGATTTCTCATAATTTATTTAATCTAAAAATGCGTATGGAAATAACACAAA
#C24        AAATAAATATGATTTCTCATAATTTATTTAATCTAAAAATGCGTATGGAAATAACACAAA
#Bur-0      AAATAAATATGATTTCTCATAATTTATTTAATCTAAAAATGCGTATGGAAATAACACAAA
#Ler        AAATAAATATGATTTCTCATAATTTATTTAATCTAAAAATGCGTATGGAAATAACACAAA
```

```
Col-0      ATTTGTTTGGATAAGATATCATCTTTTTGTCAACAAATTATATTAGTTCTAGTAAAAATA -976
Ws-0       ATTTGTTTGGATAAGATATCATCTTTTTGTCAACAAATTATATTAGTTCTAGTAAAAATA
Ct-1       ATTTGTTTGGATAAGATATCATCTTTTTGTCAACAAATTATATTAGTTCTAGTAAAAATA
Nd-1       ATTTGTTTGGATAAGATATCATCTTTTTGTCAACAAATTATATTAGTTCTAGTAAAAATA
Cvi        ATTTGTTTGGATAAGATATCATCTTTTTGTCAACAAATTATATTAGTTCTAGTAAAAATA
#Nö-0      ATTTGTTTGGATAAGATATCATCTTTTTGTCAACAAATTATATTAGTTCTAGTAAAAATA
#Oy-0      ATTTGTTTGGATAAGATATCATCTTTTTGTCAACAAATTATATTAGTTCTAGTAAAAATA
#C24       ATTTGTTTGGATAAGATATCATCTTTTTGTCAACAAATTATATTAGTTCTAGTAAAAATA
#Bur-0     ATTTGTTTGGATAAGATATCATCTTTTTGTCAACAAATTATATTAGTTCTAGTAAAAATA
#Ler       ATTTGTTTGGATAAGATATCATCTTTTTGTCAACAAATTATATTAGTTCTAGTAAAAATA

                                                    AC-rich element
Col-0      TATATAATACTAACTCTAAACGATTCAAAAAATTCAAACCAAAATAGTAGAACCTGAAAA -916
Ws-0       TATATAATACTAACTCTAAACGATTCAAAAAATTCAAACCAAAATAGTAGAACCTGAAAA
Ct-1       TATATAATACTAACTCTAAACGATTCAAAAAATTCAAACCAAAATAGTAGAACCTGAAAA
Nd-1       TATATAATACTAACTCTAAACGATTCAAAAAATTCAAACCAAAATAGTAGAACCTGAAAA
Cvi        TATATAATACTAACTCTAAACGATTCAAAAAATTCAAACCAAAATAGTAGAACCTGAAAA
#Nö-0      TATATAATACTAACTCTAAACGATTCAAAAAATTCAAACCAAAATAGTAGAACCTGAAAA
#Oy-0      TATATAATACTAACTCTAAAAGATTCAAAAAATTCAAACCAAAATAGTAGAACCTGAAAA
#C24       TATATAATACTAACTCTAAACGATTCAAAAAATTCAAACCAAAATAGTAGAACCTGAAAA
#Bur-0     TATATAATACTAACTCTAAACGATTCAAAAAATTCAAACCAAAATAGTAGAACCTGAAAA
#Ler       CATGTAATACTAACTCTAAACGATTCAAAAAATTCAAACCAAAATAGTAGAACCTGAAAA

Col-0      AATAATATCATGGTGCTAATTTATCCATTTTAAAGTATGGAAAATACGTGAAAAGTGAGG -856
Ws-0       AATAATATCATGGTGCTAATTTATCCATTTTAAAGTATGGAAAATACGTGAAAAGTGAGG
Ct-1       AATAATATCATGGTGCTAATTTATCCATTTTAAAGTATGGAAAATACGTGAAAAGTGAGG
Nd-1       AATAATATCATGGTGCTAATTTATCCATTTTAAAGTATGGAAAATACGTGAAAAGTGAGG
Cvi        AATAATATCATGGTGCTAATTTATCCATTTTAAAGTATGGAAAATACGTGAAAAGTGAGG
#Nö-0      AATAATATCATGGTGCTAATTTATCCATTTTAAAGTATGGAAAATACGTGAAAAGTGAGG
#Oy-0      AATAATATCATGGTGCTAATTTATCCATTTTAAAGTATGGAAAATACGTGAAAAGTGAGG
#C24       AATAATATCATGGTGCTAATTTATCCATTTTAAAGTATGGAAAATACGTGAAAAGTGAGG
#Bur-0     AATAATATCATGGTGCTAATTTATCCATTTTAAAGTATGGAAAATACGTGAAAAGTGAGG
#Ler       AATAATATCATGGTGCTAATTTATCCATTTTAAAGTATGGAAAATACGTGAAAAGTGAGG

Col-0      AAACAAGAAACTCCTAAATATTTTCGAAATCATCCAAGATTCCAAGTGTGTTGAGAAAGC -796
Ws-0       AAACAAGAAACTCCTAAATATTTTCGAAATCATCCAAGATTCCAAGTGTGTTGAGAAAGC
Ct-1       AAACAAGAAACTCCTAAATATTTTCGAAATCATCCAAGATTCCAAGTGTGTTGAGAAAGC
Nd-1       AAACAAGAAACTCCTAAATATTTTCGAAATCATCCAAGATTCCAAGTGTGTTGAGAAAGC
Cvi        AAACAAGAAACTCCTAAATATTTTCGAAATCATCCAAGATTCCAAGTGTGTTGAGAAGGC
#Nö-0      AAACAAGAAACTCCTAAATATTTTCGAAATCATCCAAGGTTCCAAGTGTGTTGAGAAAGC
#Oy-0      AAACAAGAAACTCCTAAATATTTTCGAAATCATCCAAGATTCCAAGTGTATTGAGAAAGC
#C24       AAACAAGAAACTCCTAAATATTTTCGAAATCATCCAAGATTCCAAGTGTGTTGAGAAAGC
#Bur-0     AAACAAGAAACTCCTAAATATTTTCGAAATCATCCAAGATTCCAAGTGTGTTGAGAAGGC
#Ler       AAACAAGAAACTCCTAAATATTTTCGAAATCATCCAAGATTCCAAGTGTGTTGAGAAGGC

Col-0      AAGTAGCTAGTTCACAGCCTGAGAGCCAATCCAAATTTTTTTTTTTTTTTTTTGAACAAC -736
Ws-0       AAGTAGCTAGTTCACAGCCTGAGAGCCAATCCAAATTTTTTTTTTTTTTTTT-GAACAAC
Ct-1       AAGTAGCTAGTTCACAGCCTGAGAGCCAATCCAAATTTTTTTTTTTTTTTTTTGAACAAC
Nd-1       AAGTAGCTAGTTCACAGCCTGAGAGCCAATCCAAATTTTTTTTTTTTTTTTTTGAACAAC
Cvi        AACTAGCTAGTTCACAGCCTGAGAGCCAATCCAAGTTTCTTACTTCATATTCTGAACAAC
#Nö-0      AAGTAGCTAGTTCACAGCCTGAGAGCCAATCCAAATTTTTTTTTTTTTTTTTTGAACAAC
#Oy-0      AAGTAGCTAGTTCACAGCCTGAGAGCCAATCCAAATTTTTTTTTTTTTTTTTTGAACAAC
#C24       AAGTAGCTAGTTCACAGCCTGAGAGCCGATCCAAATTTTTTTTTTTTTTTTTTGAACAAC
#Bur-0     AACTAGCTAGTTCACAGCCTGAGAGCCAATCCAAATTTTTTTTTTTTTTTTTTGAACAAC
#Ler       AACTAGCTAGTTCACAGCCTGAGAGCCAATCCAAATTTTTTTTTTTTTTTTTTGAACAAC
```

```
Col-0      ATAGCCAATCCAAATTCAAGTTCAATTTATAACTTATATATGGAGCTGACCAATTTGAGT -676
Ws-0       ATAGCCAATCCAAATTCAAGTTCAATTTATAACTTATATATGGAGCTGACCAATTTGAGT
Ct-1       ATAGCCAATCCAAATTCAAGTTCAATTTATAACTTATATATGGAGCTGACCAATTTGAGT
Nd-1       ATAGCCAATCCAAATTCAAGTTCAATTTATAACTTATATATGGAGCTGACCACTTTGAGT
Cvi        ATAGCCAATCCAAATTCAAGTTCAATTTATAACTTATATATGGAGCTGACCAATTTGAGT
#Nö-0      ATAGCCAATCTAAATTCAAGTTCAATTTATAACTTATATATGGAGCTGACCACTTTGAGT
#Oy-0      ATAGCCAATCCAAATTCAAGTTCAATTTATAACTTATATATGGAGCTGACCAATTTGAGT
#C24       ATAGCCAATCCAAATTCAAGTTCAATTTATAACTTATATATGGAGCTGACCAATTTGAGT
#Bur-0     ATAGCCAATCCAAATTCAAGTTCAATTTATAACTTATATATGGAGCTGCCCAATTTGAGT
#Ler       ATAGCAAATCCAAATTCAAGTTCAATTTATAACTTATATATGGAGCTGCCCAATTTGAGT

Col-0      CTTGGATTAATCCAAAGGTTCCAAATTTTTTTTCTTTTTTACAAATCTATATATATGGTC -616
Ws-0       CTTGGATTAATCCAAAGGTTCCAAATTTTTTTTCTTTTTTACAAATCTATATATATGGTC
Ct-1       CTTGGATTAATCCAAAGGTTCCAAATTTTTTTTCTTTTTTACAAATCTATATATATGGTC
Nd-1       CTTGGATTAATCCAAAGGTTCTAAATTTTTTTTCTTTTTTACAAATCTATATATATGGTC
Cvi        CTTGGATTAATCCAAAGGTTCCAAATTTTTTTTCTTTTTTACAAATCTATATATATGGTC
#Nö-0      CTTGGATTAATCCAAAGGTTCTAAATTTTTTTTCTTTTTTACAAATCTATATATATGGTC
#Oy-0      CTTGGATTAATCCAAAGGTTCCAAATTTTTTTTCTTTTTTACAAATCTATATATATGGTC
#C24       CTTGGATTAATCCAAAGGTTCCAAATTTTTTTTCTTTTTTACAAATCTATATATATGGTC
#Bur-0     CTTGGATTAATCCAAAGGTTCCAATTTTTTTTTCTTTTTTACAAATCTATATATATGGTC
#Ler       CTTGGATTAATCCAAAGGTTCCAATTTTTTTTTCTTTTTTACAAATCTATATATATGGTC

Col-0      TTCATTACAAATTTACATTACGTATTCCAATTTCTGCGGATAGATTTTTTCTTACTTCAT -556
Ws-0       TTCATTACAAATTTACATTACGTATTCCAATTTCTGCGGATAGATTTTTTCTTACTTCAT
Ct-1       TTCATTACAAATTTACATTACGTATTCCAATTTCTGCGGATAGATTTTTTCTTACTTCAT
Nd-1       TTCATTACAAATTTACATTACGTGTTCTAATTTCTGCGGATAGATTTTTTCTTACTTCAT
Cvi        TTCATTACAAATTTACATTACGTGTTCCAATTTCTGCGGATAGATTTTTTCTTACTTCAT
#Nö-0      TTCATTACAAATTTACATTACGTGTTCTAATTTCTGCGGATAGATTTTTTCTTACTTCAT
#Oy-0      TTCATTACAAATTTACATTACGTGTTCCAATTTCTGCGGATAGATTTTTTCTTACTTTAT
#C24       TTCATTACAAATTTACATTACGTGTTCCAATTTCTGCGGATAGATTTTTTCTTACTTCAT
#Bur-0     TTCATTACAAATTTACATTACGTGTTCCAATTTCTGCGGATATATTTTTTCTTACTTCAT
#Ler       TTCATTACAAATTTACATTACGTGTTCCAATTTCTGCGGATAGATTTTTTCTTACTTCAT

Col-0      ACAGAACTAAATCTAAGACCATCTCCATTGGTAGTCTCTCAAGAGAGTTTCTCACTACTA -496
Ws-0       ACAGAACTAAATCTAAGACCATCTCCATTGGTAGTCT-TCAAGAGAGTTTCTCACTACTA
Ct-1       ACAGAACTAAATCTAAGACCATCTCCATTGGTAGTCTCTCAAGAGAGTTTNTCACTACTA
Nd-1       ACAGAACTAAATCTAAGACCATCTCCATTGGTAGTCTCTCAAGAGAGTTTCTCACTACTA
Cvi        ACAGAACTAAATCTAAGACCATCTCCATTGGTAGTCTCTCAAGAGAGTTTCTCACTACTA
#Nö-0      ACAGAACTAAATCTAAGATCATCTCCATTG-TAGTCTCTCA-GAGAGTTTCTCA-TA-TA
#Oy-0      ACAGAACTAAATCTAAGACCATCTCCATTGGTAGTCTCTCAAGAGAGTTTCTCACTACTA
#C24       ACAGAACTAAATCTAAGACCATCTCCATTGGTAGTCTCTCAAGAGAGTTTCTCACTACTA
#Bur-0     ACAGAACTAAATCTAAGACCATCTCCATTGGTAGTCTCTCAAGAGAGTTTCTCACTACTA
#Ler       ACAGAACTAAATCTAAGACCATCTCCATTGGTAGTCTCTCAAGAGAGTTTCTCACTACTA

Col-0      AAATAATAAAAATAATAAAAAGAGAAGAGAGAGAGTATAGAAAGTTTCTCAAGTGAGAAA -436
Ws-0       AAATAATAAAAATAATAAAAAGAGAAGAGAGAGAGTATAGAAAGTTTCTCAAGTGAGAAA
Ct-1       AAATAATAAAAATAATAAAAAGAGAAYAGAGAGAGTATAYAAAGTTTCTCAAGTGAGAAA
Nd-1       AAATAATAAAAATAATAAAAAGAGAAGAAAGAGAGTGTAGAAAGTTTCTCAAGCGAGAAA
Cvi        AAATAATAAAAATAATAAAAAGAGAAGAGAGAGAGTATAGAAAGTTTCTCAAGTGAGAAA
#Nö-0      AAATAATAAAAATAATAAAAAGAGAAGAGAGAGAGT--AGAAAGTTTCTCAAGTGAGAAA
#Oy-0      AAATAATAAAAATAATAAAAAGAGAAGAGAGAGAGTGTAGAAAGTTTCTCAAGTGAGAAA
#C24       AAATAATAAAAATAATAAAAAGAGAAGAGAGAGAGTATAGAAAGTTTCTCAAGTGAGAAA
#Bur-0     AAATAATAAAAATAATAAAAAGAGAAGAGAGAGAGTATAGAAAGTTTCTCAAGTGAGAAA
#Ler       AAATAATAAAAATAATAAAAAGAGAAGAGAGAGAGTATAGAAAGTTTCTCAAGTGAGAAA
```

```
Col-0     CTATGAGATATTCTCTACAAGCCATCATTACATTTCTCTCCTCTCAATTGGTTCAATTTT -376
Ws-0      CTATGAGATATTCTCTACAAGCCATCATTACATTTCTCTCCTCTCAATTGGTTCAATTTT
Ct-1      CTATGAGATATTCTCTACAAGCCATCATTACATTTCTCTCCTCTCAATTGGTTCAATTTT
Nd-1      CTATGAGATACTCTCTACAAGCCATCATTACATTTCTCTCCTCTCAATTAGTTCAATTTT
Cvi       CTATGAGATATTCTCTACAAGCCATCATTACATTTCTCTCCTCTCAATTGGTTCAATTTT
#Nö-0     CTATGAGATATTCTCTACAAGCCATCATTACATTTCTCTCCTCTCAATTGGTTCAATTTT
#Oy-0     CTATGAGATACTCTCTACAAGCTATCATTACATTTCTCTCCTCTCAATTGGTTCAATTTT
#C24      CTATGAGATATTCTCTACAAGCCATCATTACATTTCTCTCCTCTCAATTGGTTCAATTTT
#Bur-0    CTATGAGATATTCTCTACAAGCCATCATTACATTTCTCTCCTCTCAATTGGTTCAATTTT
#Ler      CTATGAGATATTCTCTACAAGCCATCATTACATTTCTCTCCTCTCAATTGGTTCAATTTT

                                                             MRE1(-strand)
Col-0     TTCATTAAAATAAAAATTTAATCATATAATAAAAATTTAGTAAAATACTAATTTGTTTGT -316
Ws-0      TTCATTAAAATAAAAATTTAATCATATAATAAAAATTTAGTAAAATACTAATTTGTTTGT
Ct-1      TTCATTAAAATAAAAATTTAATCATATAATAAAAATTTAGTAAAATACTAATTTGTTTGT
Nd-1      TTCATTAAAATAAAAATTTAATCATATAATAAAAATTTAGTAAAATACTAATTTTTTTGT
Cvi       TTCATTAAAATAAAAATTTAATCATATAATAAAAATTTAGTAAAATACTAATTTGTTTGT
#Nö-0     TTCATTAAAATAAAAATTTAATCATATAATAAAAATTTAGTAAAATACTAATTTTTTTGT
#Oy-0     TTCATTAAAATAAAAATTTAATCATATAATAAAAATTTAGTAAAATACTAATTTGTTTGT
#C24      TTCATTAAAATAAAAATTTAATCATATAATAAAAATTTAGTAAAATACTAATTTGTTTGT
#Bur-0    TTCATTAAAATAAAAATTTAATCATATAATAAAAATTTAGTAAAATACTAATTTGTTTGT
#Ler      TTCATTAAAATAAAAATTTAATCATATAATAAAAATTTAGTAAAATACTAATTTGTTTGT

                    AC-rich element(-strand)       AC-rich element
Col-0     TTGAAAAACTTTTTGGGTTTCTCCTTGATGGAGATGCCCAAAGATATTTTTTACACTTCA -256
Ws-0      TTGAAAAACTTTTTGGGTTTCTCCTTGATGGAGATGCCCAAAGATATTTTTTACACTTCA
Ct-1      TTGAAAAACTTTTTGGGTTTCTCCTTGATGGAGATGCCCAAAGATATTTTTTACACTTCA
Nd-1      TTGAAAAACTTTTTGGGTTTCTCCTTGACGGAGATGCCCAAAGATATTTTTTACACTTCA
Cvi       TTGAAAAACTTTTTGGGTTTCTCCTTGATGGAGATGCCCAAAGATATTTTTTACACTTCA
#Nö-0     TTGAAAAACTTTTTGGGTTTCTCCTTGACGGAGATGCCCAAAGATATTTTTTACACTTCA
#Oy-0     TTGAAAAACTTTTTGGGTTTCTCCTTGATGGAGATGCCCAAAGATATTTTTTACACTTCA
#C24      TTGAAAAACTTTTTGGGTTTCTCCTTGATGGAGATGCCCAAAAATATTTTTTACACTTCA
#Bur-0    TTGAAAAACTTTTTGGGTTTCTCCTTGATGGAGATGCCCTAAAATATTTTTTACACTTCA
#Ler      TTGAAAAACTTTTTGGGTTTCTCCTTGATGGAGATGCCCAAAGATATTTTTTACACTTCA

                                                    AC-rich element
Col-0     TATAGTACTAATTACTAAATCTAGGATAATATATTTTTCAAACCCCAAAACAAAAATGGA -196
Ws-0      TATAGTACTAATTACTAAATCTAGGATAATATATTTTTCAAACCCCAAAACAAAAATGGA
Ct-1      TATAGTACTAATTACTAAATCTAGGATAATATATTTTTCAAACCCCAAAACAAAAATGGA
Nd-1      TATAGTACTAATTACTAAATCTAGGATAATATATTTTTCAAACCCCAAAACAAAAATGGA
Cvi       TATAGTACTAATTACTAAATCTAGGATAATATATTTTTCAGACCCCAAAACAAAAATGGA
#Nö-0     TATAGTACTAATTACTAAATCTAGGATAATATATTTTTCAAACCCCAAAACAAAAATGGA
#Oy-0     TATAGTACTAATTACTAAATCTAGGATAATATATTTTTCAAACCCCAAAACAAAAATGGA
#C24      TATAGTACTAATTACTAAATCTAGGATAATATATTTTTCAAACCCCAAAACAAAAATGGA
#Bur-0    TATAGTACTAATTACTAAATCTAGGATAATATATTTTTCAAACCCCAAAACAAAAATGGA
#Ler      TATAGTACTAATTACTAAATCTAGGATAATATATTTTTCAGACCCCCAAACAAAAATGGA

Col-0     TAGTAGTACACTACTATGCATATTTGATATTTCTATTTGTGGGAAGCGTAATATTCATTT -136
Ws-0      TAGTAGTACACTACTATGCATATTTGATATTTCTATTTGTGGGAAGCGTAATATTCATTT
Ct-1      TAGTAGTACACTACTATGCATATTTGATATTTCTATTTGTGGGAAGCGTAATATTCATTT
Nd-1      TAGTAGTACACTACTATGCATATTTGATATTTCTATTTGTGGGAAGCGTAATATTCATTT
Cvi       TAGTAGTACACTACTATGCATATTTGATATTTCTATTTGTGGGAAGCGTAATATTCATTT
#Nö-0     TAGTAGTACACTACTATGCATATTTGATATTTCTATTTGTGGGAAGCGTAATATTCATTT
#Oy-0     TAGTAGTACACTACTATGCATATTTGATATTTCTATTTGTGGGAAGCGTAATATTCATTT
#C24      TAGTAGTACACTACTATGCATATTTGATATTTCTATTTGTGGGAAGCGTAATATTCATTT
#Bur-0    TAGTAGTACACTACTATGCATATTTGATATTTCTATTTGTGGGAAGCGTAATATTCATTT
#Ler      TAGTAGTACACTACTATGCATATTTGATATTTCTATTTGTGGGAAGCGTAATATTCATTT
```

```
Col-0          CAACACGTGGTAATTCAAAAGTGGACCATCTTTCTTTTTCATCCTCACAGAAATAACACT -76
Ws-0           CAACACGTGGTAATTCAAAAGTGGACCATCTTTCTTTTTCATCCTCACAGAAATAACACT
Ct-1           CAACACGTGGTAATTCAAAAGTGGACCATCTTTCTTTTTCATCCTCACAGAAATAACACT
Nd-1           CAACACGTGGTAATTCAAAAGTGGACCATCTTTCTTTTTCATCCTCACAGAAATAACACT
Cvi            CAACACGTGGTAATTCAAAAGTGGACCATCTTTCTTTTTCATCCTCACAGAAATAACACT
#Nö-0          CAACACGTGGTAATTCAAAAGTGGACCATCTTTCTTTTTCATCCTCACAGAAATAACACT
#Oy-0          CAACACGTGGTAATTCAAAAGTGGACCATCTTTCTTTTTCATCCTCACAGAAATAACACT
#C24           CAACACGTGGTAATTCAAAAGTGGACCATCTTTCTTTTTCATCCTCACAGAAATAACACT
#Bur-0         CAACACGTGGTAATTCAAAAGTGGACCATCTTTCTTTTTCATCCTCACAGAAATAACATT
#Ler           CAACACGTGGTAATTCAAAAGTGGACCATCTTTCTTTTTCATCCTCACAGAAATAACACT

                                                         TATA-box
Col-0          GGAGGGGCAGGTGGTACAGTGAGCGAGAGACTCGTAATTTATTAATTCTAAGTTCTGACA -16
Ws-0           GGAGGGGCAGGTGGTACAGTGAGCGAGAGACTCGTAATTTATTAATTCTAAGTTCTGACA
Ct-1           GGAGGGGCAGGTGGTACAGTGAGCGAGAGACTCGTAATTTATTAATTCTAAGTTCTGACA
Nd-1           GGAGGGGCAGGTGGTACAGTGAGCGAGAGACTCGTAATTTATTAATTCTAAGTTCTGACA
Cvi            GGAGGGGCAGGTGGTACAGTGAGCGAGAGACTCGTAATTTATTAATTCTAAGTTCTGACA
#Nö-0          GGAGGGGCAGGTGGTACAGTGAGCGAGAGACTCGTAATTTATTAATTCTAAGTTCTGACA
#Oy-0          GGAGGGGCAGGTGGTACAGTGAGCGAGAGACTCGTAATTTATTAATTCTAAGTTCTGACA
#C24           GGAGGGGCAGGTGGTACAGTGAGCGAGAGACTCGTAATTTATTAATTCTAAGTTCTGACA
#Bur-0         GGAGGGGCAGGTGGTACAGTGAGCGAGAGACTCGTAATTTATTAATTCTAAGTTCTGACA
#Ler           GGAGGGGCAGGTGGTACAGTGAGCGAGAGACTCGTAATTTATTAATTCTAAGTTCTGACA

Col-0          AGCCAATAATGGAACGAAAAGAGAGAAAAAGTGTGAAAAAATGGAAAAGACTTTTGCTCT +45
Ws-0           AGCCAATAATGGAACGAAAAGAGAGAAAAAGTGTGAAAAAATGGAAAAGACTTTTGCTCT
Ct-1           AGCCAATAATGGAACGAAAAGAGAGAAAAAGTGTGAAAAAATGGAAAAGACTTTTGCTCT
Nd-1           AGCCAATAATGGAACGAAAAGAGAGAAAAAGTGTGAAAAAATGGAAAAGACTTTTGCTCT
Cvi            AGCCAATAATGGAACGAAAAGAGAGAAAAAGTGTGAAAAAATGGAAAAGACTTTTGCTCT
#Nö-0          AGCCAATAATGGAACGAAAAGAGAGAAAAAGTGTGAAAAAATGGAAAAGACTTTTGCTCT
#Oy-0          AGCCAATAATGGAACGAAAAGAGAGAAAAAGTGTGAAAAAATGGAAAAGACTTTTGCTCT
#C24           AGCCAATAATGGAACGAAAAGAGAGAAAAAGTGTGAAAAAATGGAAAAGACTTTTGCTCT
#Bur-0         AGCCAATAATGGAACGAAAAGAGAGAAAAAGTGTGAAAAAATGGAAAAGACTTTTGCTCT
#Ler           AGCCAATAATGGAACGAAAAGAGAGAAAAAGTGTGAAAAAATGGAAAAGACTTTTGCTCT
```

Figure S2: *BGLU6* promoter, alignment and putative MYB binding sites.
CLUSTALW multiple sequence alignment of *BGLU6* promoters from ten naturally *A. thaliana* accessions. Nucleotides that differs from the consensus sequence are highlighted in gray. F3GG7R-accumulating accessions are marked with #. Transcribed sequences are given in bold letters and underlined letters indicate coding sequences. Putative MYB binding sites are boxed in blue. MYB core element: CNGTTR, AC-rich element: MCCWAM, MYB related element 1 (MRE1): AMCWAMC. MYB binding sites corresponding to MYBPZM: CCWACC and MYBPLANT: MACCWAMC are not found in the *proBGLU6*.

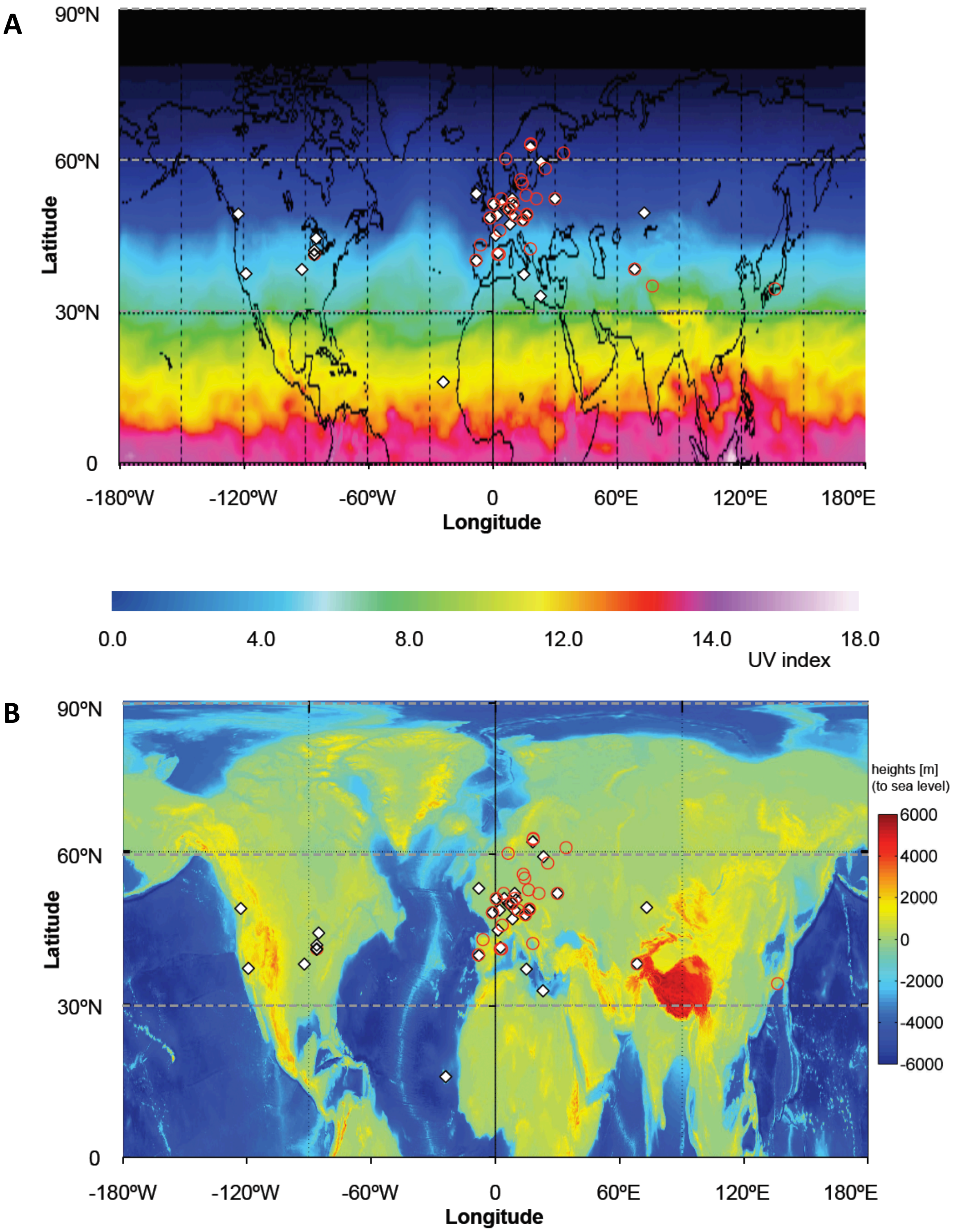

Figure S3: Correlation analyses of F3GG7R-production with enviromental factors at accession collection places.

Geographical distribution of the collection places of the *A. thaliana* Nordborg accessions in the northern hemisphere. Red circles indicate accessions which produce F3GG7R, white diamonds indicate non-producer accessions. The accession distribution map was underlaid with (A) an UV-index map (clear-sky UV index map of 12 of October 2000; http://www.temis.nl/uvradiation/info/figs/uvi20001012.gif) to indicate the level of UV radiation at the collection places and (B) with a topographic map (https://geodesy.curtin.edu.au/research/models/Earth2012/) indicating the altitude at the collection places.

```
AaAA7GT            ---MISYSLFFLLAFLFLYLVEFGISQSNAPKFS--RDDFSSEFVFGAGTLAYQYEGATA 55
DgAA7GT            --MCPSFLVTLLLLQLSSLVVVLVVWAEQLPEFNVRRDDFPSNFVFGAGTSALQVEGAIA 58
AtBGLU10_AAGT      ----------MKLYSLLSVFLVILLATSDSDAFT--RNNFPKDFLFGAATSAYQWEGAVA 48
AtBGLU6_Ler        ---------MKKTFALITIFLAFAFSGKCSDVFS--RSDFPEGFVFGSSTSAYQWEGAVA 49
DcAA5GT            MNMSCKFEIVLLVSWWLLLVLVFGVESSMFSEFD--RLDFPKHFIFGASSCAYQVEGAAF 58
                                      .: : .  .    *   * :*.. *:**:.: * * ***

AaAA7GT            EDGRSPSIWDAFTHAGGMPDKSTGDVASDGYHKYKEDVKLMSDTGLEAYRFSISWSRLLP 115
DgAA7GT            EDGKTPNIWDVDSHMGHMPDKSTTDIACDSYHRYKEDVKIMSDIGLEAYRFSIAWTRILP 118
AtBGLU10_AAGT      EDGRTPSVWDTFSHTYNRGNLGNGDITSDGYHKYKEDVKLMAEMGLESFRFSISWSRLIP 108
AtBGLU6_Ler        EDGRKPSVWDTFCHSHN--NQGNGDITCDGYHKYKEDVKLMVDTNLDAFRFSISWSRLIP 107
DcAA5GT            EDGRTLSTFDIAAHSGHL--PGNGDITSDEYHKYKEDVELMVETGLDAYRFSISWSRLIP 116
                   ***:. . :*   *       .. *::.* **:*****::* : .*:::****:*:*::*

AaAA7GT            NGRGAVNPKGIKYYNDLINELVGHGIQPHATLYHLDLPQVLEDEYEG-WLSPKIIDDFKE 174
DgAA7GT            YGRGFINPKGVEYYNNLIDTLLEHGIQPHATIYHIDHPQILEDEYGG-WLSPRMIEDFTT 177
AtBGLU10_AAGT      NGRGLINPKGLLFYKNLIKELISHGIEPHVTLYHYDLPQSLEDEYGG-WINRKIIEDFTA 167
AtBGLU6_Ler        NRRGPVNQKGLQFYKNLIQELVSHGIEPYVTLHHFDHPQYLEDEYEG-WLNHMIVEDFTA 166
DcAA5GT            NGRGPVNPKGLEYYNNLVNALLTKGTQPHVTLLHSDLPQALRDEYGGLFISPKFIDDFVA 176
                     ** :* **: :*::*:. *: :* :*:.*: * * ** *.*** * ::.  :::**

AaAA7GT            YSDVCFREFGDRVSHWTPIVEPNIVALGAYDGGQFPPQRCSYP-FGNCTAGDSTVEPYIA 233
DgAA7GT            YADVCFREFGDRVSHWTTINEPNIISLGAYDSGQIPPHRCTPPGAYNCTAGNSSVEPYKA 237
AtBGLU10_AAGT      YADVCFREFGEDVKLWTTINEATIFAIGSYDQGISPPGHCSPNKFINCTSGNSSTEPYLA 227
AtBGLU6_Ler        YADVCFREFGNHVKFWTTINEGNIFSIGGYNDGDSPPGRCS-IPGQNCLLGNSSTEPYIV 225
DcAA5GT            YADVCFREFGDRVLHWTTFNEANFLAFGDEN--------------------TPASALYLS 216
                   *:********: *  **.: * .:.::*  :                     .:   *

AaAA7GT            VHNFLLAHAAVVKLYRTKYQDIQNGWIGFNVYTNWFYPFTNSPADVEAAERVMDFMIGWI 293
DgAA7GT            MHHFLLAHASAVQIYRTKYQAKQKGLIGLNVYGFWCAPQTNSRADIEATKRATAFYTGWA 297
AtBGLU10_AAGT      GHNILLAHASASKLYKLKYKSTQKGSIGLSIFAFGLSPYTNSKDDEIATQRAKAFFYGWM 287
AtBGLU6_Ler        GHNLLLAHASVSRLYKQNYKDKQGGSIGFSILTIGFSPSTSSKDDAIATQRANDFFNGWM 285
DcAA5GT            AHHLLLAHASATRLYRENYQASQRGFIGINVYAYDFIPETNTEVDVIAAKRARDFFIGWF 276
                    *::*****:. ::*: :*:  * * **:.:      * *.:  *  *::*.  *  **

AaAA7GT            INPVVFGDYPKILKKNAGQRLPSFTKSQSEQVKGSFDFIGINHYSSAYVKDNSN-VPMP- 351
DgAA7GT            ADPLVFGDYPIIMKENVGSRLPSFTKNESELVKGSFDFIGLNHYFVFYIQDDPEEITTPI 357
AtBGLU10_AAGT      LKPLVFGDYPDEMKRTVGSRLPVFSEEESEQLKGSSDFIGIIHYTTFYVTN-KPSPSIFP 346
AtBGLU6_Ler        LGPLIYGDYPDTMKRIVGSRMPIFSEEESEQVKGSSDYIGINHYLAASITNSKLKPSISG 345
DcAA5GT            VQPLMNGEYPLTMRKNGGPRLPKFTPNETELLTGSYDFIGLNYYTAKTVKDDPVMLTVEP 336
                     *:: *:**  ::.  * *:* *: .::* :.** *:**: :*    : :     .

AaAA7GT            -DLRDFQRDMCAILTDSLNETESSQGPPTSIMSDPPGFRKILEYFKHKYNNPPIYIQENG 410
DgAA7GT            -SLRNFDSDMR--VKASVKPGDSG---------DPSGLKNLLRYFKDNYGNPPVYVHENG 405
AtBGLU10_AAGT      SMNEGFFKDMGVYMISAANSSFLL------WEATPWGLEGILEYIKQSYNNPPIYILENG 400
AtBGLU6_Ler        --NTDFYSDMNVILSFFANFSSSE------YDVAPWAIEAVLEYVKQSYGNPPVYILENG 397
DcAA5GT            ---RNYYTDQGLISSYLGNIDPYQG---HPFFNTPWGLHDVLQQFKQVYGNPPVYIHENG 390
                       .:  *         :               * .:. :*. .*. *.***:*: ***

AaAA7GT            ----FGLGVKNQVN--DTDRIDYLRDYIGSMLEAIREGSDMRGYFVWSFIDVFELLAGYQ 464
DgAA7GT            ----FGSPQNETLDD-DMGRIRYISGYIGSMLEAIKNGSDTRGYFVWSFMDAFEILSGYQ 460
AtBGLU10_AAGT      ----MPMGRDSTLQ--DTQRIEFIQAYIGAMLNAIKNGSDTRGYFVWSMIDLYELLSGYT 454
AtBGLU6_Ler        ----RPINRDSQLKEKDTPRIEFLQAYIGGVLKSIRNGSDTRGYFVWSLMDLYEIKGGYD 453
DcAA5GT            EVGDHDADYDKLIN--DIPRVEYLQGHIRAVLDAVRNGSNVKGYFVWSFLDMYELMYGTK 448
                            .. :.  *  *: ::  :* .:*.::::**: :******::* :*:  *

AaAA7GT            SGFGLYHVDFSNGNLTREPKLSAKWYSNFLKRKNDIHIQR---ADQQGISISLQ- 515
DgAA7GT            TRYGIVHVDFDDKSLKRQLKPSAQWYSNFIKKKN---------TTEDEISYSSQ- 505
AtBGLU10_AAGT      TSFGMYYVNFSDPGRKRTPKLSASWYTGFLNG-TIDVATQDTIQLQSNISGSSSL 508
AtBGLU6_Ler        VGYGLYSVNFSDPHRKRSPKLSAHWYSDFLKGKTAFLGSQGITELQSNLSPYS-- 506
DcAA5GT            FTFGLYYIDFNDPKLTRHPKLSQKWYSRFLKGEKASTKASIHTPNEAETHTYFY- 502
                     :*:  ::*.:   .*  * *  **: *::  .           :
```

Figure S4: Multiple alignment of GH1-type flavonoid glucosyltransferases.
Conserved amino acid motifs from GH1 proteins, containing catalytically active glutamate residues (grey), are boxed.

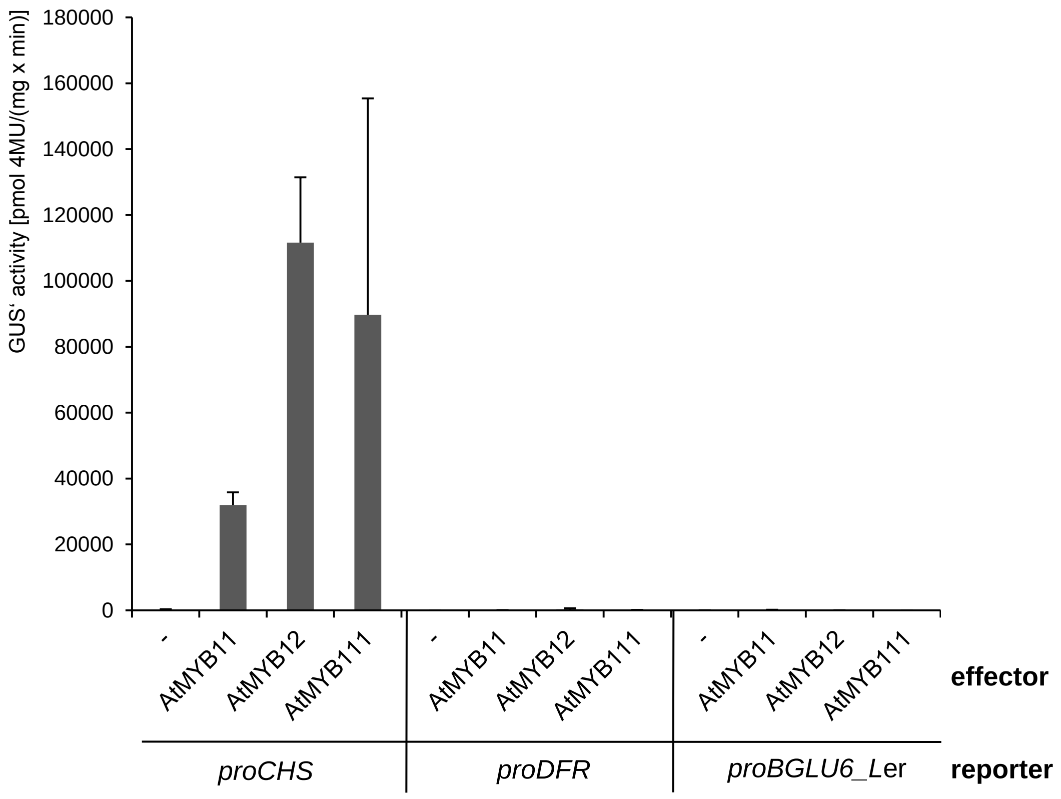

Figure S5: Co-transfection analysis of target gene specificities in *A. thaliana* At7 protoplasts.

Results from co-transfection experiments *in A. thaliana* protoplasts. The data shown are from two independent biological replicates. Promoter fragments of the *CHS* (positive control)*, DFR* (negative control) and *BGLU6* genes (reporters, fused to GUS) were assayed for their responsiveness to the 35S-driven effectors MYB11, MYB111 and MYB12, respectively. The reporter constructs for *proCHS* and *proDFR* are as described in Mehrtens *et al.* (2005). From genomic *A. thaliana* L*er* DNA a 1455 bp *BGLU6* promoter fragment was amplified using the primers RS1247 5'-GGG ACA AGT TTG TAC AAA AAA GCA GGC TTC GAA TAA ACC AAA TGA AGA AAG ACG TG-3' (containing Gateway attB1) and RS1289 5'-GGG GAC CAC TTT GTA CAA GAA AGC TGG GTC CAT GGT TTC ACA CTT TTT CTC TCT TTT CG-3' (containing Gateway attB2) and via Entry clone inserted into the Gateway-compatible vector pDISCO, resulting in a fusion to the *uidA/GUS*-ORF. Protoplast isolation and transfection experiments for the detection of transient expression were performed as described by Mehrtens *et al.* (2005). In the co-transfection experiments, a total of 25 µg of pre-mixed plasmid DNA was transfected, consisting of 10 µg of reporter plasmid, 10 µg of effector plasmid and 5 mg of the luciferase (LUC) transfection control and standardisation plasmid (*4xproUBI:LUC).* Protoplasts were incubated for 20 h at 26° C in the dark before LUC and GUS enzyme activities were measured. Specific GUS activity is given in pmol 4-MU per µg protein per min. Standardised specific GUS activity (GUS') was calculated by multiplication of the specific GUS activity value with a correction factor derived from the ratio of the specific LUC activity in the given sample to the mean specific LUC activity (describing the transformation efficiency). The figure shows GUS' activity resulting from the influence of tested effector proteins on different reporters.
